# Phylogenetic Analysis and Screening of Antimicrobial and Antiproliferative Activities of Culturable Bacteria Associated with the Ascidian* Styela clava* from the Yellow Sea, China

**DOI:** 10.1155/2019/7851251

**Published:** 2019-08-28

**Authors:** Lei Chen, Xue-Ning Wang, Chang-Ming Fu, Guang-Yu Wang

**Affiliations:** Department of Bioengineering, School of Marine Science and Technology, Harbin Institute of Technology, Weihai 264209, China

## Abstract

Over 1,000 compounds, including ecteinascidin-743 and didemnin B, have been isolated from ascidians, with most having bioactive properties such as antimicrobial, antitumor, and enzyme-inhibiting activities. In recent years, direct and indirect evidence has shown that some bioactive compounds isolated from ascidians are not produced by ascidians themselves but by their symbiotic microorganisms. Isolated culturable bacteria associated with ascidians and investigating their potential bioactivity are an important approach for discovering novel compounds. In this study, a total of 269 bacteria were isolated from the ascidian* Styela clava *collected from the coast of Weihai in the north of the Yellow Sea, China. Phylogenetic relationships among 183 isolates were determined using their 16S rRNA gene sequences. Isolates were tested for antimicrobial activity against seven indicator strains, and an antiproliferative activity assay was performed to test for inhibition of human hepatocellular carcinoma Bel 7402 and human cervical carcinoma HeLa cell proliferation. Our results showed that the isolates belonged to 26 genera from 18 families in four phyla (*Firmicutes*,* Actinobacteria*,* Proteobacteria*, and* Bacteroidetes*).* Bacillus* and* Streptomyces *were the most dominant genera; 146 strains had potent antimicrobial activities and inhibited at least one of the indicator strains. Crude extracts from 29 strains showed antiproliferative activity against Bel 7402 cells with IC_50_ values below 500 *μ*g·mL^−1^, and 53 strains showed antiproliferative activity against HeLa cells, with IC_50_ values less than 500 *μ*g·mL^−1^. Our results suggest that culturable bacteria associated with the ascidian* Styela clava *may be a promising source of novel bioactive compounds.

## 1. Introduction

Ascidians (sea squirts) are sessile marine filter-feeding invertebrates belonging to the phylum* Chordata. *Studies on ascidians can be traced back to 1847 when their blood was observed to change color following exposure to air. Investigation of this unusual phenomenon led to the isolation of a series of hydroquinoid compounds called tunichromes from the blood of several species of ascidians [1]. It was not until 1974 that the first ascidian-derived bioactive metabolite, geranyl hydroquinone, was isolated from an* Aplidium* species. This compound showed chemopreventive activity against leukemia,* Rous sarcoma* virus, and mammary carcinoma in test animals [2]. Over 1,000 compounds have been isolated from ascidians, the majority of which have bioactive properties that include antimicrobial, antitumor, and antimalarial activities [3]. Some of these compounds have been used as clinical drugs and preclinical leads [4].

Some ascidian-derived compounds, such as alkaloids, cyclic peroxides, peptides, and macrolides, have cytotoxic activities [5]. Trabectedin (also known as ecteinascidin-743 or ET-743), a milestone in the development of marine-derived drugs, was initially isolated from the Caribbean ascidian* Ecteinascidia turbinata *[6] and is of bacterial origin [7]. It was approved for the treatment of advanced soft tissue sarcoma in Europe, Russia, and South Korea and has also completed phase III randomized multicenter clinical trials for the treatment of advanced liposarcoma, leiomyosarcoma, and leiomyosarcoma [4, 8, 9].

Ascidians, together with marine sponges, corals, and other marine invertebrates, are also promising sources of novel bioactive compounds against bacterial and fungal pathogens of both humans and fish [10]. As invertebrates, ascidians rely only on innate immunity that lacks somatic recombination and long-term immune memory and has a limited array of effector responses. The efficiency of the immune system can prevent the risk of infections and select appropriate mutualistic bacterial strains for gut colonization [11]. Forazoline A, a complex and novel marine polyketide from* Actinomadura *sp. isolated from the ascidian* Ecteinascidia turbinata*, showed antifungal activity against* Candida albicans *and demonstrated* in vivo* efficacy in a disseminated candidiasis model in mice with no toxicity and was also a candidate for human trials [12, 13].

The ascidian* Styela clava *is native to the Pacific coast of Asia, ranging from the Sea of Okhotsk to Japan, Korea, and northeast China [14]. In China, it is mainly distributed in the Bohai Sea and the Yellow Sea. Recently, however, it has invaded different parts of the world's oceans, being first found in British waters in 1953, then spreading up the North Sea coast as far as Denmark and south along the Atlantic coast to Portugal [15].* S. clava* is viewed as an aggressive invader, which causes huge losses for local inshore shellfish farming [16]. However, it is beneficial to scientific research because of its rapid growth and low cost [17]. Several compounds have been isolated from* S. clava*, many of which have bioactive properties, including antihypertensive, anti-inflammatory, and antimicrobial effects [18, 19]. Two families of antimicrobial polypeptides, styelins and clavanins, were isolated from* S. clava* and identified, respectively. Styelin D showed activity against methicillin-resistant and susceptible strains of* Staphylococcus aureus* and* Pseudomonas aeruginosa *[20]. Clavanin A, which showed specific inhibition against* Escherichia coli *and* S. aureus*, is as effective as human cathelicidin LL-37 but is less toxic to humans [21].

However, collection and aquaculture of ascidians is sometimes difficult and may not be environmentally friendly, which severely limits the supply of these compounds [22]. The compounds isolated from marine invertebrates are often similar to those isolated from bacteria, which led to speculation that many of these compounds were synthesized by symbiotic bacteria rather than the animals themselves [7]. Studies on bacteria isolated from ascidians and their biological activities are relatively limited. In this study, the biodiversity of bacteria associated with* S. clava* in the Yellow Sea, China, was studied based on 16S rRNA gene sequence. Bacterial isolates were screened for potential antimicrobial and antiproliferative activities.

## 2. Materials and Methods

### 2.1. Sample Collection and Preparation

Ascidian samples (*Styela clava*) were collected from two locations of the coast of Weihai in the north of the Yellow Sea, China. One location was the intertidal zone of Xiaoshi Island, which is a sea cucumber national nature preserve (37°31′49^″^ N, 122°0′5^″^ E). The other was the surfaces of scallop farming net cages in Poyu Town (37°25′8^″^ N, 122°17′59^″^ E). After collection, the fresh samples were immediately placed into sterile plastic containers in a cooler and transported to the laboratory within 2–3 h.* S. clava* is a solitary ascidian with a leathery, bumpy, and often wrinkled outer skin. The ascidian samples were rinsed 3–5 times with filtered sterile seawater under sterile conditions to remove loosely attached microorganisms and other foreign matters from their surfaces. Following this, the* S. clava* gut was sampled using a sterile scalpel and tweezers and then homogenized.

### 2.2. Isolation of Bacterial Strains

#### 2.2.1. Isolation of Common Bacteria

Homogenates (0.5 mL) were added to sterile flasks containing 4.5 mL of sterile seawater and glass beads, shaken on a rotary shaker (180 rpm) at 28°C for 30 min, and mixed thoroughly. Following this, ten-fold serial dilutions were performed with sterile seawater, ranging from 1:100 to 1:100,000. These dilutions (100 *μ*L) were then plated on Petri dishes containing one of the 12 kinds of culture media described in Table 1. Inoculated plates were incubated at 28°C for at least 3 weeks.

#### 2.2.2. Isolation of Actinobacteria

The homogenates were heated to 55°C in a water bath for 5 min [23]. The samples were serially diluted and plated on Petri dishes containing one of the 12 kinds of culture media described in Table 1. All media were supplemented with a final concentration of 20 *μ*g nalidixic acid mL^−1^ and 100 *μ*g cycloheximide mL^−1^. The inoculated plates were incubated at 28°C for at least 3 weeks.

### 2.3. Selection and Preservation of Isolated Strains

Single colonies growing on media were selected based on colony morphology, including growth rate, shape, size, pigmentation, and margin characteristics. Following subculturing and confirmation of strain purity, the isolated bacteria were preserved in 15% glycerin at -80°C.

### 2.4. 16S rRNA Gene Sequencing and Phylogenetic Analysis

Strains were selected based on their colony morphologies on 2216E solid culture media and the results of Gram staining, and their 16S rRNA genes were sequenced to determine their phylogenetic positions. Bacteria were cultured in 2216E liquid medium, and Actinobacteria were cultured in M1 liquid media. Cells were harvested at stationary-phase by centrifugation (10,000 ×*g* for 1 min). Genomic DNA was extracted by using a Bacterial Genome DNA Extraction Kit (Sangon Biotech, China). The universal primers 27F (5'-AGAGTTTGATCCTGGCTCAG-3') and 1492R (5'-GGTTACCTTGTTACGACTT-3') were used in polymerase chain reaction (PCR) amplification of the 16S rRNA gene. Extracted genomic DNA was used as a PCR template. Genomic DNA from the bacterial strain* Escherichia coli* was used as a positive control, and sterile distilled water was used as a negative control. DNA was denatured at 94°C for 5 min, followed by 35 cycles of 94°C for 1 min, 56°C for 1 min, and 72°C for 90 s, with a final 10 min extension at 72°C. PCR products (~1,500 bp) were sequenced in Sangon Biotech, China.

Sequence similarity searches and the calculation of pairwise similarity values between isolated strains and their closely related types were performed using the EzBioCloud Database [24]. The 16S rRNA gene sequences of related strains were downloaded from the National Center for Biotechnology Information (NCBI) database (http://www.ncbi.nlm.nih.gov). Sequences were further analyzed by performing sequence alignments using Clustal X [25], with manual modification. A phylogenetic tree was constructed using the neighbor-joining method, which was implemented in the software package Molecular Evolutionary Genetics Analysis (MEGA) (version 6.0) [26]. The 16S rRNA gene sequences of the isolated bacteria were submitted to the NCBI GenBank Database under the accession numbers KT758342–KT758375, KT758393-KT758412, KT758414-KT758461, KT758463-KT758477, KT758540-KT758561, KT758563-KT758575, and KT758600-KT758630.

### 2.5. Antimicrobial Activity Screening

Antimicrobial activity was determined by observing the growth inhibition of bacteria or fungi according to the method described by Chen et al. [27]. In this study, antimicrobial activities of all 269 strains were tested by measuring inhibition zone sizes against seven indicator bacterial or fungal strains, including the Gram-negative bacteria* Escherichia coli *(ATCC 25922) and* Pseudomonas aeruginosa *(ATCC 27853), the Gram-positive bacteria* Bacillus subtilis *(ATCC 6633) and* Staphylococcus aureus *(ATCC 6538), the human pathogenic fungi* Candida albicans *(ATCC 10231), and the aquatic animal pathogenic bacteria* Vibrio parahaemolyticus *(ATCC 17802) and* Vibrio anguillarum. *Most of the indicator strains were purchased from the ATCC, except for* Vibrio anguillarum*, which was donated by Associate Professor Yuxia Zou, Institute of Oceanology, Chinese Academy of Sciences. Two* Vibrio* strains were inoculated into 2216E liquid medium and incubated at 28°C for 12 h, while the other four indicator bacteria were inoculated into peptone-beef extract liquid medium and incubated at 37°C for 12 h. The indicator fungi were inoculated into Sabouraud liquid medium (Peptone 10 g; glucose 40 g, nature seawater 1 L, pH 5.6) and incubated at 28°C for 2–3 days. The isolated bacterial strains were inoculated into 2216E solid medium at 28°C for 3–5 days, and actinobacteria were inoculated in M1 solid medium 28°C for 5–7 days. The indicator strain medium was added into 9 cm diameter Petri dishes and mixed with 0.1 mL of stationary phase cultures of the corresponding indicator bacterial suspension (or indicator fungal spore) to prepare antimicrobial assay plates. Plugs measuring 11 mm in diameter from isolated bacterial solid cultures were excised with a cork borer and placed on the surface of the corresponding indicator strain plates. Ampicillin, chloramphenicol, and norfloxacin (Sigma, St Louis, MO, USA) were used as positive controls. Disks of the same size were excised from 2216E and M1 solid medium and used in antimicrobial assays as negative controls. Then the antimicrobial assay plates were cultivated at 37°C (or 28°C) for 2–3 days for indicator bacteria or at 28°C for 5–7 days for indicator fungi. The inhibition zones around the agar plugs were measured to gauge the antimicrobial activity of the isolated bacteria. Three or more biological repeats were performed for each isolate to establish average inhibition zone sizes.

### 2.6. Preparation of Crude Extracts

All of the isolated strains were inoculated from a frozen stock into 25 mL of either 2216E liquid medium (for bacteria) or M1 liquid medium (for actinobacteria) in an Erlenmeyer flask. The cultures were incubated at 28°C for 5–7 days on a rotary shaker at 180 rpm. The culture supernatants were subsequently extracted three times with 25 mL ethyl acetate. The organic layers were separated, combined, dried over anhydrous sodium sulfate, decanted, and dried under vacuum to obtain a crude extract. The crude extract from each strain was dissolved in 1 mg dimethyl sulfoxide (DMSO) mL^−1^. The uninoculated 2216E liquid medium (for bacteria) or uninoculated M1 liquid medium (actinobacteria) was extracted and used as negative controls in the 3-(4,5-dimethylthiot the azolzyl-2-yl)-2.5-diphenyl-tetrazolium bromide (MTT) assay.

### 2.7. Antiproliferative Activity Screening

The antiproliferative activity of the crude extracts of all of the isolated strains was determined using the MTT assay as described by Chen et al. [27]. Briefly, human hepatocellular carcinoma Bel 7402 cells and human cervical carcinoma HeLa cells were purchased from the Cell Bank of the Chinese Academy of Science, China. Bel 7402 and HeLa were cultured in RPMI 1640 medium (HyClone, Thermo Fisher Scientific Inc., Logan, UT, USA) and Dulbecco's Modified Eagle Medium (DMEM) high-glucose medium (HyClone), respectively, supplemented with 10% fetal calf serum (FCS, Gibco, Grand Island, NY, USA) in 96-well microtiter plates (Corning, NY, USA). 5-Fluorouracil (5-FU, 10 ng·mL^−1^, Sigma-Aldrich, St Louis, MO, USA) was used as a positive control. The IC_50_ (50% inhibition concentration) values were defined as the concentration of an extract, which resulted in 50% growth inhibition of the tumor cells. Data are expressed as mean ± SE of three or more experiments.

## 3. Results

### 3.1. Phylogenetic Analysis of Isolated Strains

**Figure 1:**
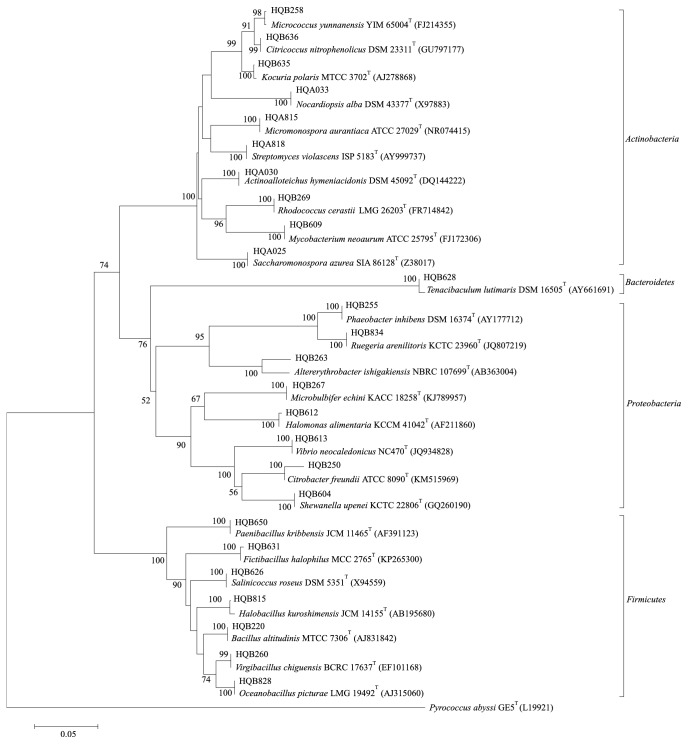
A neighbor-joining phylogenetic tree based on 16S rRNA gene sequences showing the relationships of bacterial strains associated with the ascidian* S. clava* and related taxa. A total of 26 genera were isolated from the ascidian* S. clava*, and we chose one species from each genus. Bootstrap values (>50* *%) based on 1,000 replicates are shown at branch nodes.* Pyrococcus abyssi* GE5^T^ (L19921) was used as an outgroup. Bars represent 0.05 substitutions per nucleotide position.

In this study, a total of 269 bacterial strains were isolated. A total of 125 strains were isolated from* S. clava *collected from Xiaoshi Island, and another 144 strains were isolated from* S. clava *collected from Poyu Town. Based on their colony morphologies and the results of Gram staining, a total of 183 strains were selected for phylogenetic analysis. Our 16S rRNA gene sequencing-based analysis showed that these strains belonged to 26 genera from 18 families in four phyla (Table 2) (Figure 1).

Of 183 bacterial strains, 87 were isolated from* S. clava* collected around Xiaoshi Island and were found to belong to 19 genera from 13 families in three phyla, with 16S rRNA identities in the range of 96.54%–100%. The phylum* Firmicutes* was represented by 39 strains. They were affiliated with five genera (*Bacillus*,* Fictibacillus*,* Halobacillus*,* Oceanobacillus*, and* Virgibacillus*) in one family (*Bacillaceae*), with* Bacillus* being the dominant genus. The phylum* Actinobacteria* was represented by 32 strains from seven genera (*Actinoalloteichus*,* Kocuria*,* Micrococcus*,* Micromonospora*,* Mycobacterium*,* Rhodococcus*, and* Streptomyces*) in six families (*Micrococcaceae*,* Micromonosporaceae*,* Mycobacteriaceae*,* Nocardiaceae*,* Pseudonocardiaceae*, and* Streptomycetaceae*), and* Streptomyces* was the dominant genus. The other 16 strains belonged to the* Proteobacteria*; six strains were of the *α*-*Proteobacteria*, and ten strains were of the *γ*-*Proteobacteria*. The *α*-*Proteobacteria* were distributed among three genera (*Altererythrobacter*,* Phaeobacter*, and* Ruegeria*) in two families (*Enterobacteriaceae* and* Rhodobacteraceae*), and the *γ*-*Proteobacteria* among four genera (*Halomonas*,* Microbulbifer*,* Shewanella*, and* Vibrio*) in four families (*Halomonadaceae*,* Alteromonadaceae*,* Shewanellaceae*, and* Vibrionaceae*).* Vibrio* was the dominant genus from the phylum* Proteobacteria* in this study.

The other 96 selected strains were associated with* S. clava* from Poyu Town. They belonged to 16 genera in 14 families across four phyla, with 16S rRNA gene similarities in the range of 96.72%–100%. More than half of the strains (56 strains) belong to the phylum* Firmicutes*. They were distributed among four genera (*Bacillus*,* Fictibacillus*,* Paenibacillus*, and* Salinicoccus*), from three families (*Bacillaceae*,* Paenibacillaceae*, and* Staphylococcaceae*).* Bacillus* was the dominant genus. Further 31 strains were from the phylum* Actinobacteria*. These belonged to seven genera (*Citricoccus*,* Kocuria*,* Micromonospora*,* Rhodococcus*,* Nocardiopsis*,* Saccharomonospora*, and* Streptomyces*) in six families (*Micrococcaceae*,* Micromonosporaceae*,* Nocardiaceae*,* Nocardiopsaceae*,* Pseudonocardiaceae*, and* Streptomycetaceae*). Most of these isolates were* Streptomyces. *Eight strains were from the phylum* Proteobacteria*, and all of these were *γ*-*Proteobacteria*, belonging to four genera (*Citrobacter*,* Halomonas*,* Shewanella*, and* Vibrio*) in four families (*Enterobacteriaceae*,* Halomonadaceae*,* Shewanellaceae*, and* Vibrionaceae*). Among these,* Vibrio* was the dominant genus. A single strain, HQB628, was from the phylum* Bacteroidetes* and belonged to the genus* Tenacibaculum* of the family* Flavobacteriaceae*.

In this study, the bacterial diversity associated with the ascidian species* S. clava *collected from different locations exhibited similar high-abundance bacteria and also exhibited differences in low-abundance bacteria. Among a total of 26 genera isolated during our study, nine genera (*Kocuria*,* Micromonospora*,* Rhodococcus*,* Streptomyces*,* Bacillus*,* Fictibacillus*,* Halomonas*,* Shewanella*, and* Vibrio*) were shared by two ascidian samples from different locations (Table 2). The* S. clava *collected from both locations was associated with a larger proportion of bacteria of the genera* Bacillus* and* Streptomyces*. A total of 27 species represented the genus* Bacillus*, and 14 species represented the genus* Streptomyces*, with 16S rRNA gene sequence similarities in the range of 96.69%–100.00% (data listed in the Supplementary Material Tables [Supplementary-material supplementary-material-1] and [Supplementary-material supplementary-material-1]). Ten genera were only isolated from ascidian samples collected at Xiaoshi Island, including three genera in the* Actinobacteria*, three genera in the* Firmicute*, three genera in the *α*-*Proteobacteria*, and one genus in the *γ*-*Proteobacteria*. Seven genera were only isolated from ascidian samples collected at Poyu Town, including three genera from the* Actinobacteria*, one genus from the* Bacteroidetes*, two genera from the* Firmicute*, and one genus from the *γ*-*Proteobacteria*.

Three strains, HQB252, HQB272, and HQB233, had a sequence similarity of only 96.85% to* Bacillus thermotolerans*, 96.54% to* Kocuria rosea,* and 96.90% to* Bacillus cereus*, respectively. They did not cluster with any validly named species, suggesting that they are potentially novel species of the genus* Bacillus *and* Kocuria*.

### 3.2. Antimicrobial Activity of Isolated Strains

Antimicrobial activity screening tests showed that of the 269 bacterial strains tested 146 strains (54.28% of all strains) exhibited antimicrobial activities against at least one of the indicator strains used. A total of 27 strains (10.04% of all strains) inhibited at least three of the indicator strains (Table 3). One isolate, HQA032 (*Streptomyces* sp.), displayed inhibitory activity against six indicator bacterial strains.

Isolated strains exhibited higher antimicrobial activity against Gram-positive bacteria (*B. subtilis *and* S. aureus*) than against Gram-negative bacteria (*E. coli*,* P. aeruginosa*,* V. parahaemolyticus*, and* V. anguillarum*), with 117 strains (43.49%) inhibiting Gram-positive bacteria, while there were 40 strains (14.87%) inhibiting Gram-negative bacteria. A total of 98 inhibited* B. subtilis*. Among them, 25 strains had inhibition zones of more than 16 mm. Two strains HQA032 and HQA046 (both are* Streptomyces *sp.) had the strongest inhibitory effects against* B. subtilis*, with inhibition zone diameters of 22 mm and 27 mm, respectively. Activity against* S. aureus* was found in 67 strains (24.91% strains), especially strain HQA046 (*Streptomyces* sp.), which had an inhibition zone of 26 mm. However, low antimicrobial activity was found against Gram-negative bacteria. Only 12 strains inhibited the growth of* E. coli*, and only four strains showed activity against* P. aeruginosa*. As for antimicrobial activity against marine animal bacterial pathogens, 11 strains displayed activities against* V. Parahaemolyticus*, and 25 strains against* V. anguillarum*. In addition, 45 strains (16.73% strains) displayed activities against the human pathogenic fungus* C*.* albicans*, with two strains, HQA030 (*Actinoalloteichus *sp.) and HQA819 (*Streptomyces *sp.), both having inhibition zone diameters of more than 22 mm. These results suggested that some culturable strains associated with* S. clava*, especially actinobacterial strains, could be promising sources for the treatment of infected diseases.

### 3.3. Antiproliferative Activity of Isolated Strains

The MTT assay was used to evaluate the ability of crude extracts of the isolated strains to inhibit the proliferation of human hepatocellular carcinoma Bel 7402 cells and human cervical carcinoma HeLa cells. Our results showed that, among the 269 strains, the extracts of 187 strains (69.52% strains) showed antiproliferative activity against Bel 7402 cells, with 29 extracts (10.78%) showing high antiproliferative activity, with IC_50_ below 500 *μ*g·mL^−1^ (Table 4). In particular, the extract of HQB237 (*Bacillus* sp.) displayed strong antiproliferative activity against Bel 7402, with an IC_50_ of 0.44 *μ*g·mL^−1^.

The extracts from 208 strains (77.32% strains) showed antiproliferative activity against HeLa cells, with 53 strains (19.70% strains) having an IC_50_ lower than 500 *μ*g·mL^−1^ (Table 5). Five extracts significantly suppressed the proliferation of HeLa cells with IC_50_ values less than 100 *μ*g·mL^−1^. These strains were as follows: HQA806 (*Streptomyces* sp.), HQB281 (*Rhodococcus* sp.), HQB287 (*Bacillus* sp.), HQB824 (*Bacillus* sp.), and HQB827 (*Bacillus *sp.). Of these, the extract of HQA806 showed the strongest antiproliferative activity against HeLa cells, with an IC_50_ of 25.88 *μ*g·mL^−1^.

## 4. Discussion

Because of their high biodiversity and chemodiversity, marine bacteria are considered a promising source of novel drugs. In recent years, the richness and diversity of microbial communities associated with ascidians have surprised many investigators. The research methods employed in their study have included both culture-dependent and culture-independent techniques, with the latter, including DNA fingerprinting techniques, metagenomic libraries, and the latest next-generation sequencing techniques [28].

To date, bacterial strains representing at least 34 genera belonging to 23 families in 5 phyla (*Actinobacteria*,* Bacteroidetes*,* Firmicutes*,* Proteobacteria*, and* Verrucomicrobia*) have been isolated from ascidians [10]. These include strains of* Acinetobacter*,* Bacillus*,* Halomonas*,* Kocuria*,* Microbulbifer*,* Micrococcus*,* Ruegeria*,* Shewanella*, and* Streptomyces*, among others. Some strains isolated from ascidians represent novel genera or novel species, including* Ascidiaceibacter salegens*,* Halomonas halocynthiae*,* Labilibacter aurantiacus*,* Ruegeria halocynthiae*,* Streptomyces hyaluromycini*, and* Tenacibaculum halocynthiae *[29–34]. The culture-dependent approach applied in this study resulted in the isolation of 269 strains distributed across 26 genera from 18 families in 4 phyla* Actinobacteria*,* Bacteroidetes*,* Firmicutes*, and* Proteobacteria* (*α*-*Proteobacteria* and *γ*-*Proteobacteria*).* Bacillus* and* Streptomyces *were the dominant genera. Of the genera found in our study, 15 genera have been previously isolated from both* S. clava* and other kinds of ascidians. These genera are* Bacillus*,* Halobacillus*,* Halomonas*,* Kocuria*,* Microbulbifer*,* Micrococcus*,* Micromonospora*,* Nocardiopsis*,* Paenibacillus*,* Ruegeria*,* Shewanella*,* Streptomyces*,* Tenacibaculum*,* Vibrio*, and* Virgibacillus* [10, 29–32]. A total of 11 genera,* Actinoalloteichus*,* Altererythrobacter*,* Citricoccus*,* Citrobacter*,* Fictibacillus*,* Mycobacterium*,* Oceanobacillus*,* Phaeobacter*,* Rhodococcus*,* Saccharomonospora*, and* Salinicoccus*, have not yet been isolated from ascidians using culture-dependent methods. The genera* Actinoalloteichus*,* Citricoccus*,* Mycobacterium*,* Rhodococcus*, and* Saccharomonospora *are rare* Actinobacteria* but have been isolated from marine environments previously [35–37].* Actinoalloteichus* species were previously isolated from the soil in the cold desert [38], the rhizosphere of fig trees [39], and marine sponges [40]. Members of the* Citricoccus* genus were previously isolated from marine sponges [41], marine macroalgae [42], and marine sediments [43]. Some species of this genus such as* Citricoccus nitrophenolicus* use aromatic compounds as the only source of carbon and energy [44], and* C. nitrophenolicus *may show lipolytic activity at low temperatures [45]. The genus* Altererythrobacter*, belonging to the phylum* Proteobacteria*, has been isolated from various marine and terrestrial environments such as deep-sea water [46], tidal flats [47], marine sediments [48], forests, desert soil [49, 50], hot springs [51], and the air [52]. The genus* Fictibacillus*, belonging to the phylum* Firmicutes*, has been previously isolated from different environments, including hot springs [53], industrial wastes [54], metal ores [55], and marine sediments [56]. Our study increased the knowledge of the diversity of available ascidian-derived microorganisms, providing new resources useful for the screening of novel strains and bioactive compounds.

Our* S. clava* ascidian samples were collected from two locations, Xiaoshi Island and Poyu Town. The bacterial diversity associated with the ascidian species* S. clava* collected from different locations had similarities in high-abundance bacteria but also had differences in low-abundance bacteria. On the one hand, nine genera were shared by ascidian samples from both locations, and there were 69 strains (79.31% of 87 sequenced isolates from Xiaoshi Island), and 88 strains (91.67% of 96 sequenced isolates from Poyu Town) belonged to these nine genera, respectively. Among them, there are a larger proportion of bacteria belonged to two genera (*Bacillus *and* Streptomyces*). Strains belonging to the genus* Bacillus* were the greatest in number, with 39.08% (34 strains) and 54.17% (52 strains) of all the sequenced isolates from the two locations, respectively, and then followed by the genus* Streptomyces*, which accounted for 24.14% (21 strains) and 25.00% (24 strains) of the sequenced strains isolated from the two locations, respectively. These two genera are common in the marine environment and have been frequently isolated from marine animals [57]. On the other hand, the ascidian-derived strains from low-abundance genera collected at the two locations were different. Ten genera, including 18 strains (20.69%), were only isolated from ascidian samples collected from Xiaoshi Island, and seven genera (nearly half of the 16 genera), including eight strains (8.33%), were only isolated from ascidian samples collected at Poyu Town. Our results suggest that there might be a stable symbiotic relationship between ascidians and these high-abundance bacteria. In another study that investigated gut bacterial diversity in the ascidian* Ciona intestinalis*, the results showed that samples from three geographically isolated populations revealed a striking similarity in the abundant operational taxonomic unit (OTUs). These shared OTUs between disparate populations mask the effects of their highly variable environments [58]. Bacterial and chemical analysis of 32 different ascidians from a broad expanse of the tropical Pacific Ocean showed that ascidian-derived microorganisms were highly diverse, and this diversity does not correlate with geographical location or latitude. Ascidian-derived microorganisms were stable over time and space. The majority of these microorganisms are species-specific. Location-specific bacteria are found in low abundance in the ascidians and mostly represent widespread strains. Such species may be associated with changes in seawater or environment, and their specific association with ascidians is uncertain [59].

To date, 150 natural products have been isolated from ascidian-derived microorganisms, and many of them, such as polyketides, peptides, alkaloids, terpenoids, and other types, have been found to have antimicrobial activities [10]. Two new lipopeptides, peptidolipins B and E, which were isolated from an actinobacterium* Nocardia *sp. associated with the ascidian* Trididemnum orbiculatum*, showed antibacterial activities against both methicillin-resistant* Staphylococcus aureus *(MRSA) and methicillin-sensitive* Staphylococcus aureus* (MSSA) [60]. Arenimycin, which was isolated from an ascidian-derived actinobacterium strain* Salinispora arenicola*, exhibited potent antimicrobial activities against drug-resistant* Staphylococci*, one* Mycobacterium* strain, and a variety of other Gram-positive microorganisms [61]. The development of multiple resistances among pathogenic microorganisms has become a public health problem, and the discovery of novel and efficient antimicrobial compounds has, therefore, become increasingly important. In this study, antimicrobial activities of bacteria associated with the ascidian* Styela clava *were screened for the first time. More than half of the strains show antimicrobial activity and represent a potential source for novel active compounds. Most of the active strains showed inhibition against Gram-positive indicator bacteria, with comparatively fewer strains inhibiting Gram-negative bacteria. Similar results have previously been obtained from other ascidians, as well as other marine invertebrates including corals and sponges [62–64]. Gram-negative bacteria, as a consequence of their possessing an outer membrane barrier and multidrug efflux pumps, are typically more resistant to antibiotics than Gram-positive bacteria [65, 66]. The outer membrane barrier in Gram-negative bacteria, which comprises a lipid bilayer impermeable to large, charged molecules, limits the intracellular access of various antibiotic classes [67]. Multidrug efflux pumps are tripartite membrane-localized transport proteins capable of transporting a wide range of compounds, including drug molecules, out of cells, thus preventing the access of the drug to its target [68]. Nevertheless, we found several strains displaying potent antimicrobial activities against Gram-negative* V. anguillarum* and* V. parahaemolyticus*, including the two* Bacillus *strains HQB224 and HQB229. These results suggest that the ascidian* S. clava* is a potential source of antibiotic-producing bacteria. Studies are ongoing in our laboratory to identify the bioactive compounds responsible for the observed effects.

Ascidians are prominent sources of novel compounds with antiproliferative activities, including the well-known anticancer agents, didemnin B and ET-743, which have been proven to be of bacterial origin [7, 69]. To date, a quarter of all compounds isolated from ascidian-derived microorganisms have been shown to possess antiproliferative activity [70, 71]. In this study, crude extracts from 129 out of the 269 isolated strains showed more than 50% growth inhibition in Bel 7402 or HeLa cells on treatment at 1 mg·mL^−1^ (data listed in the Supplementary Material Tables [Supplementary-material supplementary-material-1] and [Supplementary-material supplementary-material-1]). More than half of the 129 strains (51.94% of the strains) showed both antimicrobial and antiproliferative activities. Compared to extracts from four* Bacillus* strains and one* Streptomyces* strain that significantly suppressed the proliferation of Bel 7402 or HeLa cells with IC_50_ values less than 100 *μ*g·mL^−1^, the extract of one* Rhodococcus* strain HQB281 showed stronger antiproliferative activity against HeLa cells, with an IC_50_ of 61.74 *μ*g·mL^−1^. The genus* Rhodococcus* has been isolated from a broad range of environments including marine animals [72]. With a broad catabolic diversity and an array of unique enzymatic activities, the genus* Rhodococcus* exhibits significant potential for environmental and biotechnological applications including fossil fuel biodesulfurization, bioactive steroid production, and large-scale production of acrylamide and acrylic acid [73, 74]. However, there are limited studies on the antiproliferative activity of metabolites from the* Rhodococcus* strain. A particular* Rhodococcus* strain isolated from polluted soil was found to exhibit antiproliferative activity against two human cancer cell lines, hepatocellular carcinoma HepG2 and cervical carcinoma HeLa cells, with an IC_50_ of 73.39 and 33.09 *μ*g·mL^−1^, respectively [73]. In this study, the antiproliferative activities of extracts from the* Fictibacillus* and* Phaeobacter* strains have been reported for the first time.

In this study, bacterial ascidian-derived strains from the genus* Bacillus* had the highest antimicrobial and antiproliferative activities, supporting the hypothesis that they might play a protective role in their hosts [75].* Bacillus *strains are widely distributed in marine environments and are found to be associated with marine animals [76, 77]. They are culturable on many general-purpose media and have high proliferation rates and great adaptability [75]. Many secondary metabolites have been isolated from* Bacillus*, including peptides, terpenoids, polyketides, and isocoumarins [78]. These diverse compounds exhibit a wide range of biological properties, including antimicrobial, anticancer, and algicidal activities [79]. However, as fewer marine* Bacillus* strains have been examined for pharmaceutical activities, more studies are required.

*Streptomyces *is another dominant bacterial genus with bioactive activity found in this study. Some* Streptomyces* strains, such as strain HQA806 and HQA802, showed potent antiproliferative activity against both Bel 7402 and HeLa and showed antimicrobial activity against several indicator strains. Marine* Streptomyces* produce many well-known bioactive compounds, including the capoamycin-type antibiotic dioxamycin [80], the streptogramin etamycin [81], and the thiopeptide antibiotic nosiheptide [82]. Moreover, a great number of new molecules were obtained from marine* Streptomyces*, for example, lobophorins [83], polyene acids [80], and polycyclic xanthones [84]. Most compounds showed potent inhibition of common indicator strains, and some of them displayed anti-MRSA activity [85].

Rare actinobacteria, known as non-*Streptomyces*, are less frequently isolated than the common* Streptomyces* strains, even though this trend may not be reflected in the abundance of these strains in their ecological [86]. Rare actinobacteria are a promising source with potential novel metabolites of pharmaceutical relevance. Diverse new rare species, including novel genera and novel families of* Actinobacteria*, have been isolated from marine habitats (coastal, tidal, and deep-sea sediments), marine animals (sponges, corals, and ascidians), seawater, and mangrove forests [64, 85–88]. In our study, some rarely isolated actinobacteria showed potent antimicrobial activity. For example, three bacterial strains from the genus* Micromonospora* displayed inhibitory activity against* E. coli*,* B. subtilis*,* S. aureus*, and* C. albicans*. The genus* Micromonospora* can be easily found in marine invertebrates such as sponges [89].* Micromonospora* is considered one of the most prolific producers of secondary metabolites among the* Actinobacteria*, which, as a group, display a rich chemical diversity and various pharmaceutically and medically relevant bioactivities [90]. HQA030, belonging to the genus* Actinoalloteichus*, showed potent antimicrobial activity in our study. Two bioactive compounds, F1 (hydrophilic) and F2 (hydrophobic), were isolated from an* Actinoalloteichus* strain, which was isolated from a saline Saharan soil and showed antibacterial and antifungal activities against a broad spectrum of microorganisms known to be human and plant pathogens [91]. Further studies will be necessary to identify the bioactive substances produced by HQA030.

Bacterial strains with a 16S rRNA gene sequence similarity of less than 97% are considered to be separate species [92]. Recently, a similarity of 98.6% in the 16S rRNA gene sequence was suggested as a threshold for differentiating two species [93]. In our study, the strains HQB252, HQB272, and HQB233 shared similarities of less than 97% with other known species and may, therefore, be potentially new species of the genus* Bacillus *or* Kocuria*. To characterize these potentially new species, we will perform a polyphasic taxonomic study.

## 5. Conclusions

Our study reveals the diversity of bacteria associated with the ascidian* S. clava* and reports a broad spectrum of antimicrobial and antiproliferative activities displayed by these strains. Our results suggest that the culturable bacteria associated with the ascidian* S. clava *may constitute a promising source of novel bioactive compounds.

## Figures and Tables

**Table 1:** Compositions of the media for the isolation of bacteria associated with ascidian *Styela clava*.

Media	Composition
2216E	Peptone 5 g; yeast extract 1 g; FePO_4_ 0.01 g; agar 18 g; natural seawater 1 L; pH 7.6.
M1	Soluble starch 10 g; yeast extract 4 g; peptone 2 g; agar 18 g; natural seawater 750 mL; deionized water 250 mL.
M2	Glycerol 6 mL; arginine 1 g; K_2_HPO_4_·3H_2_O 1 g; MgSO_4_·7H_2_O 0.5 g; agar 18 g; natural seawater 1 L.
M3	L-asparagine 0.1 g; casein peptone 2 g; K_2_HPO_4_·3H_2_O 0.05 g; MgSO_4_·7H_2_O 0.1 g; FeSO_4_·7H_2_O 0.01 g; agar 18 g; nature seawater 1 L; pH 7.0.
M4	Yeast extract 5 g; L- asparagine 1 g; glycerol 10 mL; K_2_HPO_4_·3H_2_O 1 g; KNO_3_ 5 g; agar 18 g; nature seawater 1 L; pH 7.5-8.5.
M5	Agar 18 g; natural seawater 1 L.
M8	Aspartic acid 0.1 g; casein peptone 2 g; sodium propionate 4 g; K_2_HPO_4_·3H_2_O 0.05 g; MgSO_4_·7H_2_O 0.1 g; FeSO_4_·7H_2_O 0.01 g; agar 18 g; natural seawater 1 L; pH 7.0.
M12	Soluble starch 10 g; casein 0.5 g; K_2_HPO_4_·3H_2_O 0.5 g; NaCl 20 g; deionized water 1 L; agar 18 g; pH 7.0.
M13	Yeast extract 4 g; soluble starch 15 g; K_2_HPO_4_·3H_2_O 1 g; MgSO_4_·7H_2_O 0.5 g; agar 18 g; natural seawater 1 L; pH 7.0.
M18	Glucose 10 g; asparagine 1 g; K_2_HPO_4_·3H_2_O 1 g; FeSO_4_·7H_2_O 0.001 g; ZnSO_4_·7H_2_O 0.01 g; agar 18 g; natural seawater 750 mL; deionized water 250 mL; pH 7.0.
M19	Glycerol 10 g; acid hydrolyzed casein 0.3 g; KNO_3_ 2 g; NaC1 2 g; K_2_HPO_4_·3H_2_O 2 g; MgSO_4_·7H_2_O 0.05 g; FeSO_4_·7H_2_O 0.01 g; CaCO_3_ 0.2 g; agar 18 g; natural seawater 750 mL; deionized water 250 mL; pH 7.0.
Peptone–beef extract medium	Beef extract 5.0 g; peptone 10.0 g; NaCl 5.0 g; agar 18 g; nature seawater 1 L; pH 7.4-7.6.

**Table 2:** Identification of the 183 bacterial strains isolated from *S. clava* collected from two different locations (Xiaoshi Island and Poyu Town) based on the similarities of the 16S rRNA gene sequences.

Phylum	Genus	Number of isolates from Xiaoshi Island	Number of isolates from Poyu Town
*Actinobacteria*	*Citricoccus*	0	1
	*Micrococcus*	4	0
	*Kocuria*	1	1
	*Micromonospora*	2	1
	*Mycobacterium*	1	0
	*Rhodococcus*	2	1
	*Nocardiopsis*	0	2
	*Actinoalloteichus*	1	0
	*Saccharomonospora*	0	1
	*Streptomyces*	21	24
*Bacteroidetes*	*Tenacibaculum*	0	1
*Firmicutes*	*Bacillus*	34	52
	*Fictibacillus*	1	2
	*Halobacillus*	2	0
	*Oceanobacillus*	1	0
	*Virgibacillus*	1	0
	*Paenibacillus*	0	1
	*Salinicoccus*	0	1
*Proteobacteria* (the class *α*-*Proteobacteria*)	*Altererythrobacter*	1	0
	*Phaeobacter*	1	0
	*Ruegeria*	4	0
*Proteobacteria* (the class *γ*-*Proteobacteria*)	*Citrobacter*	0	1
	*Halomonas*	2	2
	*Microbulbifer*	2	0
	*Shewanella*	1	1
	*Vibrio*	5	4
Total number		87	96

**Table 3:** Antimicrobial activities of the isolated strains associated with ascidian *Styela clava*.

Test strains	Inhibition degree against indicator strains						
	Ec	Pa	Bs	Sa	Ca	Va	Vp
HQA013	-	+	+	-	-	++	++
HQA014	-	-	+++	-	-	++	++
HQA022	-	-	++	+	+	-	-
HQA029	++	-	++	+	-	-	-
HQA030	-	-	++	++	++++	-	-
HQA032	+	+	++++	+++	-	++	+
HQA034	-	-	+++	++	+	+	-
HQA046	-	-	++++	++++	-	+++	++
HQA051	-	-	++	+	+	-	-
HQA053	-	-	+++	+	+	-	-
HQA056	-	-	+++	++	+++	-	-
HQA057	-	-	++	+	+	-	-
HQA058	-	-	+++	++	++	-	-
HQA802	-	-	+++	++	+	++	-
HQA809	-	-	+++	+++	+	++	-
HQA811	-	-	++	+	-	++	-
HQA819	-	-	+	++	++++	-	-
HQB224	+	-	-	+	-	-	+++
HQB255	-	-	+	+	-	++	-
HQB268	+	+	+	+	-	-	-
HQB288	-	-	+	+	+	-	-
HQB296	-	-	+	++	++	+	-
HQB603	-	-	+	+++	+	-	-
HQB610	+	-	+	+	-	-	-
HQB613	++	-	++	++	-	++	-
HQB636	++	-	+++	++	-	+++	-
HQB641	-	-	+	+	+	++	-

Only the bacterial strains with antimicrobial activity against at least three indicator strains were listed.

Antimicrobial activities were tested against *Escherichia coli* (Ec), *Pseudomonas aeruginosa* (Pa), *Bacillus subtilis* (Bs), *Staphylococcus aureus* (Sa), *Candida albicans* (Ca), *Vibrio anguillarum* (Va), and *Vibrio parahaemolyticus* (Vp).

Symbols of inhibition degree against indicator strains: (-), no inhibition; (+), 11 < inhibition zone < 13 mm; (++), 13 ≤ inhibition zone < 16 mm; (+++), 16 ≤ inhibition zone < 22 mm; (++++), inhibition zone ≥ 22 mm.

**Table 4:** Antiproliferative activities against Bel 7402 cells of crude extracts from isolated bacterial strains associated with the ascidian *Styela clava *by MTT assay.

Test strains	IC_50_ (*μ*g·mL^−1^)		Test strains	IC_50_ (*μ*g·mL^−1^)
HQA008	177.22±12.51		HQB268	331.7±5.11
HQA018	356.35±3.32		HQB270	127.61±11.66
HQA038	355.55±7.54		HQB292	264.28±3.28
HQA054	391.01±5.24		HQB606	120.38±4.26
HQA802	432.97±6.21		HQB646	180.78±6.23
HQA810	183.24±7.17		HQB650	343.61±7.49
HQB216	295.46±8.92		HQB653	179.73±3.57
HQB237	0.44±0.09		HQB667	291.69±11.34
HQB243	161.06±12.54		HQB670	400.92±4.50
HQB246	187.83±10.28		HQB811	374.7±3.41
HQB251	417.54±4.03		HQB823	86.77±5.92
HQB252	263.21±3.10		HQB824	191.61±11.22
HQB255	276.44±6.31		HQB827	282.84±12.23
HQB265	165.87±7.62		HQF015	321.95±6.42
HQB266	414.39±4.67			

Only the bacterial strains with antiproliferative activity IC_50_⩽500 *μ*g·mL^−1^ were listed.

Data is expressed as mean ± SE of three or more experiments.

**Table 5:** Antiproliferative activities against HeLa cells of crude extracts from isolated strains associated with the ascidian* Styela clava* by MTT assay.

Test strains	IC_50_ (*μ*g·mL^−1^)	Test strains	IC_50_ (*μ*g·mL^−1^)
HQA018	471.42±4.43	HQB293	122.05±1.68
HQA024	390.18±8.15	HQB295	450.54±3.72
HQA037	346.96±8.27	HQB299	348.12±6.84
HQA802	422.5±3.11	HQB602	248.85±4.28
HQA806	25.88±3.57	HQB603	376.95±5.76
HQA809	160.44±5.97	HQB606	109.82±7.61
HQB218	206.47±3.69	HQB620	453.36±3.02
HQB221	150.78±10.23	HQB624	104.04±4.29
HQB223	181.03±5.14	HQB627	254.68±3.86
HQB225	288.1±5.22	HQB628	339.46±6.85
HQB226	267.55±4.21	HQB646	113.97±7.32
HQB227	458.09±6.33	HQB650	218.64±7.19
HQB230	260.71±7.25	HQB653	213.13±4.55
HQB231	155.03±5.45	HQB663	308.37±4.03
HQB232	205.97±4.86	HQB666	207.91±5.25
HQB239	298.21±4.11	HQB667	129.77±5.28
HQB242	295.53±6.25	HQB805	332.63±4.37
HQB244	261.23±7.77	HQB811	338.56±6.32
HQB246	187.35±6.25	HQB813	278.38±7.74
HQB255	204.81±10.25	HQB823	193.10±8.31
HQB266	302.2±3.12	HQB824	55.84±7.80
HQB267	346.18±7.29	HQB825	202.05±7.36
HQB268	373.57±6.27	HQB827	42.57±5.68
HQB279	150.01±11.23	HQB828	193.42±7.38
HQB281	61.74±4.50	HQB835	158.29±12.23
HQB287	66.25±8.37	HQB837	265.57±8.52
HQB292	101.32±7.54		

Only the bacterial strains with antiproliferative activity IC_50_⩽500 *μ*g·mL^−1^ were listed.

Data is expressed as mean ± SE of three or more experiments.

## Data Availability

The data used to support the findings of this study are included within the article.

## Supplementary Materials

Supplementary Materials Table S1: The comparison of 16S rRNA, antimicrobial activities, and antiproliferative activities of 125 strains associated with the ascidian* Styela clava* collected from Xiaoshi Island. Table S2: The comparison of 16S rRNA, antimicrobial activities, and antiproliferative activities of 144 strains associated with the ascidian* Styela clava* collected from Poyu Town.
