# Supplementary material for: Phylogenetic Analysis and Screening of Antimicrobial and Antiproliferative Activities of Culturable Bacteria Associated with the Ascidian Styela clava from the Yellow Sea, China
Source: Biomed Res Int. 2019 Aug 28;2019:7851251. doi: 10.1155/2019/7851251 (PMC6735190; doi:10.1155/2019/7851251)
Supplement: Supplementary Materials — Table S1: The comparison of 16S rRNA, antimicrobial activities, and antiproliferative activities of 125 strains associated with the ascidian Styela clava collected from Xiaoshi Island. Table S2: The comparison of 16S rRNA, antimicrobial activities, and antiproliferative activities of 144 strains associated with the ascidian Styela clava collected from Poyu Town. [file 7851251.f1.docx]

TABLE S1: The comparison of 16S rRNA, antimicrobial activities, and antiproliferative activities of 125 strains associated with the ascidian *Styelaclava* collected from Xiaoshi Island

| **Test strains** | **16S rRNA comparison results and similarity (%)** | **Inhibition degree against indicator strains^a^** | | | | | | | **Antiproliferative activities IC_50_ (μg**·**mL^-1^) ^b^** | |
| --- | --- | --- | --- | --- | --- | --- | --- | --- | --- | --- |
|  |  | **Ec** | **Pa** | **Bs** | **Sa** | **Ca** | **Va** | **Vp** | **Bel 7402** | **HeLa** |
| HQA027 | *Streptomyces massiliensis* AP10^T^ (JX101691) (97.94) | - | - | - | - | ++ | - | - | - | 739.47±8.39 |
| HQA028 | *Streptomyces lusitanus* NBRC 13464^T^ (AB184424) (98.06) | - | - | +++ | + | - | - | - | - | - |
| HQA029 | *Micromonospora citrea* DSM 43903^T^ (AB184424) (99.01) | ++ | - | ++ | + | - | - | - | - | - |
| HQA030 | *Actinoalloteichus hymeniacidonis* DSM 45092^T^ (CP014859) (98.45) | - | - | ++ | ++ | ++++ | - | - | - | 508.88±4.89 |
| HQA801 | *Streptomyces hydrogenans* NBRC 13475^T^ (AB184868) (100) | - | - | + | - | - | - | - | - | - |
| HQA802 | *Streptomyces hydrogenans* NBRC 13475^T^ (AB184868) (99.53) | - | - | +++ | ++ | + | ++ | - | 432.97±6.21 | 422.5±3.11 |
| HQA803 | *Streptomyces gougerotii* NBRC 3198^T^ (AB184742) (99.36) | - | - | +++ | ++ | - | - | - | 825.53±9.26 | - |
| HQA804 | *Streptomyces gougerotii* NBRC 3198^T^ (AB184742) (99.90) | - | - | + | - | - | - | - | 798.38±8.28 | - |
| HQA805 | *Streptomyces gougerotii* NBRC 3198^T^ (AB184742) (99.39) | - | - | ++ | + | - | - | - | - | - |
| HQA806 | *Streptomyces gougerotii* NBRC 3198^T^ (AB184742) (100) | - | - | - | +++ | + | - | - | 530.63±7.26 | 25.88±3.57 |
| HQA807 | *Streptomyces hydrogenans* NBRC 13475^T^ (AB184868) (99.23) | - | - | ++ | ++ | - | - | - | 607.71±6.32 | 657.60±6.27 |
| HQA808 | *Streptomyces gougerotii* NBRC 3198^T^ (AB184742) (99.36) | - | - | ++ | + | - | - | - | - | - |
| HQA809 | *Streptomyces coelicoflavus* NBRC 15399^T^ (AB184650) (99.55) | - | - | +++ | +++ | + | ++ | - | 668.03±7.37 | 160.44±5.97 |
| HQA810 | *Streptomyces violascens* ISP 5183^T^ (AY999737) (99.71) | - | - | - | - | - | - | - | 183.24±7.17 | 982.38±8.32 |
| HQA811 | *Streptomyces violascens* ISP 5183^T^ (AY999737) (99.64) | - | - | ++ | + | - | ++ | - | 775.87±11.76 | - |
| HQA812 | *Streptomyces violascens* ISP 5183^T^ (AY999737) (99.80) | - | - | +++ | ++ | - | - | - | - | - |
| HQA813 | *Streptomyces coelicoflavus* NBRC 15399^T^ (AB184650) (100) | - | - | - | - | - | - | - | - | 862.35±7.24 |
| HQA814 | *Streptomyces violascens* ISP 5183^T^ (AY999737) (99.28) | - | - | ++ | - | - | - | - | - | - |

TABLE S1 continued

| **Test strains** | **16S rRNA comparison results and similarity (%)** | **Inhibition degree against indicator strains^a^** | | | | | | | **Antiproliferative activities IC_50_ (μg**·**mL^-1^) ^b^** | |
| --- | --- | --- | --- | --- | --- | --- | --- | --- | --- | --- |
|  |  | **Ec** | **Pa** | **Bs** | **Sa** | **Ca** | **Va** | **Vp** | **Bel 7402** | **HeLa** |
| HQA815 | *Micromonospora aurantiaca* ATCC 27029^T^ (NR074415) (99.91) | - | - | - | +++ | + | - | - | 657.31±9.37 | - |
| HQA816 | *Streptomyces gougerotii* NBRC 3198^T^ (AB184742) (99.89) | - | - | ++ | + | - | - | - | - | - |
| HQA817 | *Streptomyces hydrogenans* NBRC 13475^T^ (AB184868) (99.68) | - | - | ++ | - | - | - | - | - | - |
| HQA818 | *Streptomyces violascens* ISP 5183^T^ (AY999737) (100) | - | - | ++ | - | - | - | - | - | - |
| HQA819 | *Streptomyces violascens* ISP 5183^T^ (AY999737) (96.69) | - | - | + | ++ | ++++ | - | - | - | - |
| HQA820 | *Streptomyces prasinopilosus* NRRL B-2711^T^ (EF626597) (97.38) | - | - | - | - | - | - | - | - | - |
| HQB255 | *Phaeobacter inhibens* DSM 16374^T^ (AY177712) (99.41) | - | - | + | + | - | ++ | - | 276.44±6.31 | 204.81±10.25 |
| HQB257 | *Vibrio hyugaensis* LMG 28466^T^ (LC004912) (99.80) | - | - | - | - | - | - | - | - | - |
| HQB258 | *Micrococcus yunnanensis* YIM 65004^T^ (FJ214355) (99.90) | - | - | - | - | - | - | - | 527.44±7.16 | - |
| HQB259 | Unidentified | - | - | - | - | - | - | - | - | 536.12±5.15 |
| HQB260 | *Virgibacillus chiguensis* BCRC 17637^T^ (EF101168) (99.90) | - | - | +++ | + | - | - | - | - | 852.57±7.47 |
| HQB261 | Unidentified | - | - | - | - | + | - | - | - | 646.87±6.32 |
| HQB263 | *Altererythrobacter ishigakiensis* NBRC 107699^T^ (AB363004) (97.73) | - | - | - | - | - | - | - | - | 838.37±8.52 |
| HQB264 | Unidentified | - | - | - | - | - | - | - | - | - |
| HQB265 | *Bacillus siamensis* KCTC 13613^T^ (KT781674) (99.48) | - | - | - | - | - | - | - | 165.87±7.62 | - |
| HQB266 | Unidentified | - | - | + | ++ | - | - | - | 414.39±4.67 | 302.2±3.12 |
| HQB267 | *Microbulbifer echini* KACC 18258^T^ (KJ789957) (99.44) | - | - | - | + | - | - | - | - | 346.18±7.29 |

TABLE S1 continued

| **Test strains** | **16S rRNA comparison results and similarity (%)** | **Inhibition degree against indicator strains^a^** | | | | | | | | | | | **Antiproliferative activities IC_50_ (μg**·**mL^-1^) ^b^** | | | | |  |
| --- | --- | --- | --- | --- | --- | --- | --- | --- | --- | --- | --- | --- | --- | --- | --- | --- | --- | --- |
|  |  | **Ec** | **Pa** | **Bs** | **Sa** | | **Ca** | | **Va** | | **Vp** | | **Bel 7402** | | **HeLa** | | |  |
| HQB269 | *Rhodococcus cerastii* LMG 26203^T^ (FR714842) (99.90) | - | - | - | | - | | - | | - | | - | | - | | - | | |
| HQB270 | Unidentified | - | - | - | | - | | - | | - | | - | | 127.61±11.66 | | - | | |
| HQB271 | *Bacillus licheniformis* ATCC 14580^T^ (KY034369) (98.83) | - | - | - | | - | | - | | - | | - | | - | | - | | |
| HQB272 | *Kocuria rosea* DSM 20447^T^ (MG871404) (96.54) | - | - | - | | - | | - | | - | | - | | - | | - | | |
| HQB273 | Unidentified | - | - | - | | + | | + | | - | | - | | - | | - | | |
| HQB274 | Unidentified | - | - | - | | - | | - | | - | | + | | - | | - | | |
| HQB275 | Unidentified | - | - | - | | - | | - | | - | | - | | - | | - | | |
| HQB276 | Unidentified | - | - | - | | - | | - | | - | | - | | - | | 738.73±7.43 | | |
| HQB277 | *Bacillus tequilensis* KCTC 13622^T^ (LC038165) (98.57) | - | - | - | | - | | - | | - | | - | | 893.58±7.25 | | - | | |
| HQB278 | Unidentified | - | - | + | | - | | - | | - | | - | | - | | - | | |
| HQB279 | Unidentified | - | - | - | | - | | - | | - | | - | | - | | 150.01±11.23 | | |
| HQB280 | *Bacillus tequilensis* KCTC 13622^T^ (LC038165) (98.53) | - | - | - | | - | | - | | - | | - | | - | | - | | |
| HQB281 | *Rhodococcus corynebacterioides* DSM 20151^T^ (AF430066) (99.67) | - | - | - | | - | | - | | - | | - | | - | | 61.74±4.50 | | |
| HQB282 | *Bacillus infantis* NRRL B-14911^T^ (JF495108) (99.13) | ++ | - | - | | - | | - | | - | | - | | - | | - | | |
| HQB283 | *Vibrio hepatarius* LMG 20362^T^ (AJ345063) (99.82) | - | - | - | | ++ | | + | | - | | - | | 925.63±9.37 | | - | | |
| HQB284 | Unidentified | - | - | + | | - | | - | | - | | - | | - | | - | | |
| HQB285 | *Bacillus paralicheniformis* KACC 18426^T^ (KY694465) (99.81) | - | - | - | | - | | - | | - | | - | | - | | - | | |
| HQB286 | *Bacillus altitudinis* MTCC 7306^T^ (AJ831842) (99.11) | - | - | - | | - | | - | | - | | - | | - | | | - | |
| HQB287 | *Bacillus qingshengii* JCM 19454^T^ (JX293295) (99.26) | - | - | - | | - | | - | | - | | - | | - | | | 66.25±8.37 | |

TABLE S1 continued

| **Test strains** | **16S rRNA comparison results and similarity (%)** | **Inhibition degree against indicator strains^a^** | | | | | | | | | | | **Antiproliferative activities IC_50_ (μg**·**mL^-1^) ^b^** | | | | | |  |  |
| --- | --- | --- | --- | --- | --- | --- | --- | --- | --- | --- | --- | --- | --- | --- | --- | --- | --- | --- | --- | --- |
|  |  | **Ec** | **Pa** | **Bs** | **Sa** | | **Ca** | | **Va** | | **Vp** | | **Bel 7402** | | | **HeLa** | | |  |  |
| HQB288 | Unidentified | - | - | + | | + | | + | | - | | - | | - | | | | - | |  |
| HQB289 | *Bacillus megaterium* NBRC 15308^T^ (AB271751) (98.92) | - | - | - | | - | | - | | ++ | | - | | - | | | | - | |  |
| HQB290 | *Bacillus paralicheniformis* KACC 18426^T^ (KY694465) (99.82) | - | - | - | | - | | - | | - | | - | | - | | | | 762.33±9.62 | |  |
| HQB291 | Unidentified | - | - | - | | - | | ++ | | - | | - | | - | | | | 973.73±8.72 | |  |
| HQB292 | Unidentified | - | - | - | | - | | - | | - | | - | | 264.28±3.28 | | | | 101.32±7.54 | |  |
| HQB293 | *Bacillus altitudinis* MTCC 7306^T^ (AJ831842) (99.02) | - | - | - | | - | | - | | + | | - | | - | | | | 122.05±1.68 | |  |
| HQB294 | Unidentified | - | - | - | | - | | - | | - | | - | | - | | | | - | |  |
| HQB295 | *Bacillus siamensis* KCTC 13613^T^ (KT781674) (98.76) | - | - | - | | - | | - | | - | | - | | - | | | | 450.54±3.72 | |  |
| HQB296 | Unidentified | - | - | + | | ++ | | ++ | | + | | - | | - | | | | - | |  |
| HQB297 | Unidentified | - | - | - | | - | | - | | - | | - | | - | | | | - | |  |
| HQB298 | *Ruegeria arenilitoris* KCTC 23960^T^ (JQ807219) (99.23) | - | - | + | | - | | - | | ++ | | - | | - | | | | - | |  |
| HQB299 | Unidentified | - | - | - | | - | | - | | - | | - | | - | | | | 348.12±6.84 | |  |
| HQB601 | Unidentified | - | - | - | | - | | - | | - | | - | | - | | | | 968.35±7.32 | |  |
| HQB602 | Unidentified | - | - | - | | ++ | | - | | - | | - | | 983.82±9.82 | | | | 248.85±4.28 | |  |
| HQB603 | Unidentified | - | - | + | | +++ | | + | | - | | - | | - | | | | 376.95±5.76 | |  |
| HQB604 | *Shewanella upenei* KCTC 22806^T^ (GQ260190) (99.41) | - | - | + | | + | | - | | - | | - | | - | | | | - | |  |
| HQB605 | *Vibrio neocaledonicus* NC470^T^ (JQ934828) (99.15) | - | - | - | | - | | - | | - | | - | | - | | | | - | |  |
| HQB606 | Unidentified | - | - | - | | +++ | | ++ | | - | | - | | 120.38±4.26 | | | | 109.82±7.61 | |  |
| HQB607 | Unidentified | - | - | +++ | | ++ | | - | | - | | - | | | - | | - | | | |
| HQB608 | *Ruegeria arenilitoris* KCTC 23960^T^ (JQ807219) (99.02) | - | - | - | | - | | - | | - | | - | | | - | | - | | | |

TABLE S1 continued

| **Test strains** | **16S rRNA comparison results and similarity (%)** | **Inhibition degree against indicator strains^a^** | | | | | | | | | | | **Antiproliferative activities IC_50_ (μg**·**mL^-1^) ^b^** | | | | |  |  |
| --- | --- | --- | --- | --- | --- | --- | --- | --- | --- | --- | --- | --- | --- | --- | --- | --- | --- | --- | --- |
|  |  | **Ec** | **Pa** | **Bs** | **Sa** | | **Ca** | | **Va** | | **Vp** | | **Bel 7402** | | **HeLa** | | |  |  |
| HQB609 | *Mycobacterium neoaurum* ATCC 25795^T^ (FJ172306) (99.45) | - | - | - | | - | | - | | - | | - | | - | | - | | | |
| HQB610 | *Vibrio hyugaensis* LMG 28466^T^ (LC004912) (98.74) | + | - | + | | + | | - | | - | | - | | - | | - | | | |
| HQB611 | *Bacillus vietnamensis* JCM 11124^T^ (AB099708) (98.67) | - | - | - | | - | | - | | - | | - | | - | | 683.85±8.53 | | | |
| HQB612 | *Halomonas alimentaria*KCCM 41042^T^ (AF211860) (99.62) | - | - | - | | - | | - | | - | | - | | 784.55±9.56 | | 751.85±11.03 | | | |
| HQB613 | *Vibrio neocaledonicus*NC470^T^ (JQ934828) (99.90) | ++ | - | ++ | | ++ | | - | | ++ | | - | | - | | - | | | |
| HQB614 | *Halomonas litopenaei* SYSU ZJ2214^T^ (KP301091) (99.36) | - | - | - | | - | | - | | - | | - | | - | | 684.92±7.29 | | | |
| HQB615 | Unidentified | - | - | + | | - | | - | | - | | - | | - | | 632.67±5.28 | | | |
| HQB616 | Unidentified | - | - | - | | - | | - | | - | | - | | - | | 858.43±8.23 | | | |
| HQB801 | *Micrococcus yunnanensis* YIM 65004^T^ (FJ214355) (99.60) | - | - | - | | - | | - | | - | | - | | - | | - | | | |
| HQB802 | *Bacillus altitudinis* MTCC 7306^T^ (AJ831842) (99.02) | - | - | - | | - | | - | | - | | - | | - | | - | | | |
| HQB803 | Unidentified | ++ | - | ++ | | - | | - | | - | | - | | - | | - | | | |
| HQB804 | *Bacillus altitudinis* MTCC 7306^T^ (AJ831842) (98.49) | - | - | ++ | | + | | - | | - | | - | | - | | - | | | |
| HQB805 | Unidentified | - | - | - | | + | | + | | - | | - | | - | | 332.63±4.37 | | | |
| HQB806 | *Bacillus paramycoides* LMG 28876T^T^ (KJ812444) (99.53) | - | - | - | | - | | - | | - | | - | | - | | - | | | |
| HQB807 | Unidentified | - | - | ++ | | - | | - | | - | | - | | - | | - | | | |
| HQB808 | *Bacillus velezensis* CECT 5686^T^ (AY603658) (99.15) | - | - | - | | - | | - | | - | | - | | - | | 789.39±5.58 | | | |
| HQB809 | *Bacillus oceanisediminis* JCM 16506^T^ (GQ292772) (99.36) | - | - | - | | - | | - | | - | | - | | - | | | - | |  |
| HQB810 | *Bacillus sonorensis* NBRC 101234^T^ (NR_113993) (99.50) | - | - | - | | - | | - | | - | | - | | - | | | - | |  |
| HQB811 | Unidentified | - | - | - | | - | | - | | ++ | | - | | 374.7±3.41 | | | 338.56±6.32 | |  |
| HQB812 | Unidentified | - | - | +++ | | - | | - | | - | | - | | - | | | - | |  |

TABLE S1 continued

| **Test strains** | **16S rRNA comparison results and similarity (%)** | **Inhibition degree against indicator strains^a^** | | | | | | | | | | | | | **Antiproliferative activities IC_50_ (μg**·**mL^-1^) ^b^** | | | | |  |  |
| --- | --- | --- | --- | --- | --- | --- | --- | --- | --- | --- | --- | --- | --- | --- | --- | --- | --- | --- | --- | --- | --- |
|  |  | **Ec** | **Pa** | | **Bs** | | **Sa** | | **Ca** | | **Va** | | **Vp** | | **Bel 7402** | | **HeLa** | | |  |  |
| HQB813 | Unidentified | - | | - | | - | | ++ | | - | | +++ | | - | | - | | | 278.38±7.74 | | |
| HQB814 | *Bacillus licheniformis* ATCC 14580^T^(AE017333) (99.21) | - | | - | | - | | - | | - | | - | | - | | - | | | - | | |
| HQB815 | *Halobacillus kuroshimensis* JCM 14155^T^ (AB195680) (99.56) | - | | - | | + | | - | | - | | - | | - | | - | | | - | | |
| HQB816 | *Bacillus indicus* LMG 22858^T^ (KF791344) (99.32) | - | | - | | - | | - | | - | | - | | - | | - | | | - | | |
| HQB817 | *Bacillus cereus* ATCC 14579^T^ (AE016877) (99.11) | - | | - | | - | | - | | - | | - | | - | | - | | | - | | |
| HQB818 | *Bacillus infantis* NRRL B-14911^T^ (JF495108) (99.42) | - | | - | | - | | - | | - | | - | | - | | - | | | - | | |
| HQB819 | *Bacillus oryzaecorticis* KACC 17217^T^ (KF548480) (100) | - | | - | | - | | - | | - | | - | | - | | - | | | 805.02±3.49 | | |
| HQB820 | *Bacillus jeotgali* KCCM 41040^T^ (AF221061) (99.61) | - | | - | | + | | - | | ++ | | - | | - | | - | | | - | | |
| HQB821 | *Bacillus licheniformis* ATCC 14580^T^ (AE017333) (99.23) | - | | - | | - | | - | | - | | - | | - | | - | | | - | | |
| HQB822 | *Bacillus altitudinis* MTCC 7306^T^ (AJ831842) (99.64) | - | | - | | - | | - | | - | | - | | - | | - | | | - | | |
| HQB823 | Unidentified | - | | - | | - | | - | | ++ | | - | | - | | 86.77±5.92 | | | 193.10±8.31 | | |
| HQB824 | *Bacillus siamensis* KCTC 13613^T^ (KT781674) (99.01) | - | | - | | - | | ++ | | ++ | | - | | - | | 191.61±11.22 | | | 55.84±7.80 | | |
| HQB825 | Unidentified | - | | - | | + | | - | | - | | - | | - | | - | | | 202.05±7.36 | | |
| HQB826 | Unidentified | - | | - | | + | | - | | - | | - | | - | | - | | | - | | |
| HQB827 | *Bacillus velezensis* CECT 5686^T^ (AY603658)(98.17) | - | | - | | - | | - | | - | | - | | - | | 282.84±12.23 | | | 42.57±5.68 | | |
| HQB828 | *Oceanobacillus picturae* LMG 19492^T^ (AJ315060) (99.33) | - | | - | | - | | - | | - | | - | | - | | - | | | 193.42±7.38 | | |
| HQB829 | Unidentified | - | | ++ | | - | | - | | - | | - | | - | | - | | | 524.77±6.39 | | |
| HQB830 | Unidentified | - | | - | | - | | - | | - | | - | | - | | - | | | 693.58±3.67 | | |
| HQB831 | *Micrococcus yunnanensis* YIM 65004^T^ (FJ214355) (99.7) | - | | - | | - | | - | | - | | - | | - | | - | | | 848.58±6.62 | | |
| HQB832 | *Bacillus aryabhattai* MTCC 7902^T^ (EF114313) (99.60) | - | | - | | - | | - | | - | | - | | - | | - | | - | | |  |
| HQB833 | *Halobacillus kuroshimensis* JCM 14155^T^ (AB195680) (99.03) | - | | - | | - | | - | | - | | - | | - | | - | | - | | |  |

TABLE S1 continued

| **Test strains** | **16S rRNA comparison results and similarity (%)** | **Inhibition degree against indicator strains^a^** | | | | | | | | | | | | | **Antiproliferative activities IC_50_ (μg**·**mL^-1^) ^b^** | | | |  |
| --- | --- | --- | --- | --- | --- | --- | --- | --- | --- | --- | --- | --- | --- | --- | --- | --- | --- | --- | --- |
|  |  | **Ec** | **Pa** | | **Bs** | | **Sa** | | **Ca** | | **Va** | | **Vp** | | **Bel 7402** | | **HeLa** | |  |
| HQB834 | *Ruegeria arenilitoris* KCTC 23960^T^ (JQ807219) (99.4) | - | | - | | - | | - | | - | | - | | - | | - | | 729.83±7.37 | |
| HQB835 | *Bacillus velezensis* CECT 5686^T^ (AY603658)(99.32) | - | | - | | - | | - | | - | | - | | - | | 928.45±4.32 | | 158.29±12.23 | |
| HQB836 | *Ruegeria atlantica* CECT 4292^T^ (AF124521) (99.43) | - | | - | | + | | - | | - | | - | | - | | - | | - | |
| HQB837 | *Bacillus hwajinpoensis* KCCM 41641^T^ (AF541966) (98.75) | - | | - | | - | | - | | - | | - | | - | | - | | 265.57±8.52 | |
| HQB838 | *Bacillus vietnamensis* JCM 11124^T^ (AB099708) (99.05) | - | | - | | - | | - | | - | | - | | - | | - | | - | |
| HQB839 | *Micrococcus yunnanensis* YIM 65004^T^ (FJ214355) (99.73) | - | | - | | - | | - | | - | | - | | - | | - | | - | |
| HQB840 | *Bacillus flexus* NBRC 15715^T^ (FJ584305) (99.02) | - | | - | | - | | - | | - | | - | | - | | - | | - | |
| HQB841 | *Fictibacillus halophilus* MCC 2765^T^ (KP265300) (99.80) | - | | - | | - | | - | | - | | - | | - | | - | | - | |
| HQB842 | *Microbulbifer echini* KACC 18258^T^ (KJ789957) (98.20) | - | | - | | - | | - | | - | | - | | - | | - | | - | |
| HQF015 | Unidentified | - | | - | | + | | + | | - | | - | | - | | 321.95±6.42 | | 714.38±8.39 | |

^a^ Antimicrobial activities were tested against *Escherichia coli* (Ec), *Pseudomonas aeruginosa* (Pa), *Bacillus subtilis* (Bs), *Staphylococcus aureus* (Sa), *Candida albicans* (Ca), *Vibrio anguillarum* (Va) and *Vibrio parahaemolyticus* (Vp).

Symbols of inhibition degree against indicator strains: (-), no inhibition; (+), 11 < inhibition zone < 13 mm; (++), 13 ≤ inhibition zone < 16 mm; (+++), 16 ≤ inhibition zone < 22 mm; (++++), inhibition zone ≥ 22 mm.

^b^ Data is expressed as mean ± SE of three or more experiments. The symbol (-) means that the crude extract of test strains at a concentration of 1 mg·mL^-1^ showed less than 50% growth inhibition of the tumor cells.

TABLE S2: The comparison of 16S rRNA, antimicrobial activities, and antiproliferative activities of 144 strains associated with the ascidian *Styelaclava* collected from

Poyu Town

| **Test strains** | **16S rRNA comparison results and similarity (%)** | | **Inhibition degree against indicator strains^a^** | | | | | | | | | | **Antiproliferative activities IC_50_ (μg**·**mL^-1^) ^b^** | |  |
| --- | --- | --- | --- | --- | --- | --- | --- | --- | --- | --- | --- | --- | --- | --- | --- |
|  |  |  | **Ec** | | **Pa** | | | **Bs** | **Sa** | **Ca** | **Va** | **Vp** | **Bel 7402** | **HeLa** |  |
| HQA008 | *Streptomyces albidoflavus* DSM 40455^T^ (Z76676) (99.64) | | - | | - | | | + | - | - | - | - | 177.22±12.51 | 947.13±7.99 |  |
| HQA009 | Unidentified | | - | | - | | | - | - | - | - | - | - | - |  |
| HQA010 | *Streptomyces violascens* ISP 5183^T^ (AY999737) (98.01) | | - | | - | | | + | - | - | - | - | - | - |  |
| HQA011 | *Bacillus kochii* WCC 4582^T^ (FN995265) (99.63) | | - | | - | | | - | - | - | - | - | - | 550.34±8.24 |  |
| HQA012 | Unidentified | | - | | - | | | - | - | - | - | - | - | 968.56±9.82 |  |
| HQA013 | *Streptomyces violascens* ISP 5183^T^ (AY999737) (97.44) | | - | | + | | | + | - | - | ++ | ++ | - | 656.36±7.84 |  |
| HQA014 | Unidentified | | - | | - | | | +++ | - | - | ++ | ++ | - | 878.48±8.76 |  |
| HQA015 | *Streptomyces pratensis* NRRL B-24916^T^ (JQ806215) (99.64) | | - | | - | | | - | - | - | ++ | - | - | - |  |
| HQA016 | *Streptomyces violascens* ISP 5183^T^ (AY999737) (97.79) | | - | | - | | | - | - | - | ++ | - | - | 891.89±6.26 |  |
| HQA017 | *Streptomyces albidoflavus* DSM 40455^T^ (Z76676) (99.63) | | - | | - | | | - | - | - | - | - | - | - |  |
| HQA018 | *Streptomyces violascens* ISP 5183^T^ (AY999737) (98.65) | | - | | - | | | - | - | - | - | - | 356.35±3.32 | 471.42±4.43 |  |
| HQA019 | *Bacillus altitudinis* MTCC 7306^T^ (AJ831842) (98.34) | | - | | - | | | - | - | - | - | - | - | 698.35±6.19 |  |
| HQA020 | *Streptomyces coelicoflavus* NBRC 15399^T^ (AB184650) (98.53) | | - | | - | | | + | - | - | + | - | 970.34±7.48 | 792.56±9.10 |  |
| HQA021 | Unidentified | | - | | - | | | - | - | - | - | - | - | - |  |
| HQA022 | *Streptomyces hydrogenans* NBRC 13475^T^ (AB184868) (99.81) | | - | | - | | | ++ | + | + | - | - | - | - |  |
| HQA023 | *Streptomyces hydrogenans* NBRC 13475^T^ (AB184868) (100) | | - | | - | | | ++ | - | - | - | - | - | - |  |
| HQA024 | *Streptomyces albogriseolus* NRRL B-1305^T^ (AJ494865) (100) | | - | | - | | | ++ | +++ | - | - | - | - | 390.18±8.15 |  |
| HQA025 | *Saccharomonospora azurea* SIA 86128^T^ (Z38017) (99.44) | | - | | - | | | ++ | - | - | - | - | - | 692.34±6.82 |  |
| HQA026 | *Streptomyces gougerotii* NBRC 3198^T^ (AB184742) (99.79) | | - | | - | | | + | - | - | - | - | - | - |  |
| HQA031 | | *Streptomyces gougerotii* NBRC 3198^T^ (AB184742) (99.68) | | - | | - | +++ | | ++ | - | - | - | 853.27±7.36 | - |  |

TABLE S2 continued

| **Test strains** | **16S rRNA comparison results and similarity (%)** | | **Inhibition degree against indicator strains^a^** | | | | | | | | | | | | | | **Antiproliferative activities IC_50_ (μg**·**mL^-1^) ^b^** | | | |
| --- | --- | --- | --- | --- | --- | --- | --- | --- | --- | --- | --- | --- | --- | --- | --- | --- | --- | --- | --- | --- |
|  |  |  | **Ec** | | **Pa** | | **Bs** | | **Sa** | | **Ca** | | **Va** | | **Vp** | | **Bel 7402** | | **HeLa** | |
| HQA032 | *Streptomyces griseoincarnatus* LMG19316^T^ (AJ781321) (99.88) | + | | + | | ++++ | | +++ | | - | | ++ | | + | | 785.54±9.43 | | 695.79±7.83 | |  |
| HQA033 | *Nocardiopsis alba* DSM 43377^T^ (X97883) (99.89) | - | | - | | + | | - | | - | | - | | - | | - | | - | |  |
| HQA034 | *Streptomyces violascens* ISP 5183^T^ (AY999737) (98.96) | - | | - | | +++ | | ++ | | + | | + | | - | | - | | - | |  |
| HQA035 | Unidentified | - | | - | | +++ | | - | | + | | - | | - | | 743.78±8.30 | | - | |  |
| HQA036 | Unidentified | - | | - | | - | | - | | - | | - | | - | | - | | 745.62±5.12 | |  |
| HQA037 | Unidentified | - | | - | | +++ | | + | | - | | - | | - | | - | | 346.96±8.27 | |  |
| HQA038 | Unidentified | - | | - | | ++ | | + | | - | | - | | - | | 355.55±7.54 | | 948.43±6.91 | |  |
| HQA039 | Unidentified | - | | - | | +++ | | - | | - | | - | | - | | 783.63±8.145 | | - | |  |
| HQA040 | *Streptomyces hainanensis* YIM 47672^T^ (AM398645) (99.56) | - | | - | | - | | - | | + | | - | | - | | - | | - | |  |
| HQA041 | Streptomyces gougerotii NBRC 3198^T^ (AB184742) (98.46) | - | | - | | +++ | | - | | - | | - | | - | | - | | - | |  |
| HQA042 | *Nocardiopsis alba* DSM 43377^T^ (X97883) (98.92) | - | | - | | - | | - | | - | | - | | - | | - | | - | |  |
| HQA043 | Unidentified | - | | - | | +++ | | ++ | | - | | - | | - | | - | | - | |  |
| HQA044 | *Streptomyces diastaticus* subsp. *ardesiacus* NBRC 15402^T^ (AB184653) (98.86) | - | | - | | +++ | | - | | - | | - | | - | | - | | - | |  |
| HQA045 | *Streptomyces albogriseolus* NRRL B-1305^T^ (AJ494865) (99.52) | - | | - | | + | | - | | - | | - | | - | | - | | - | |  |
| HQA046 | *Streptomyces tunisiensis* DSM 42037^T^ ( HQ822265) (98.92) | - | | - | | ++++ | | ++++ | | - | | +++ | | ++ | | 759.18±7.65 | | - | |  |
| HQA047 | *Streptomyces coeruleorubidus* ISP 5145^T^ (AJ306622) (99.13) | - | | - | | +++ | | - | | + | | - | | - | | - | | - | |  |
| HQA048 | Unidentified | - | | - | | | - | - | | - | | - | | - | | 771.60±6.13 | | - | |  |
| HQA049 | Unidentified | - | | - | | | +++ | - | | + | | - | | - | | - | | - | |  |

TABLE S2 continued

| **Test strains** | **16S rRNA comparison results and similarity (%)** | | **Inhibition degree against indicator strains^a^** | | | | | | | | | | | | | **Antiproliferative activities IC_50_ (μg**·**mL^-1^) ^b^** | | | |
| --- | --- | --- | --- | --- | --- | --- | --- | --- | --- | --- | --- | --- | --- | --- | --- | --- | --- | --- | --- |
|  |  |  | **Ec** | | **Pa** | **Bs** | | **Sa** | | **Ca** | | **Va** | | **Vp** | | **Bel 7402** | | **HeLa** | |
| HQA050 | *Micromonospora wenchangensis* DSM 45709^T^ (JQ768361) (99.18) | - | | - | | + | + | | - | | - | | - | | - | | - | |  |
| HQA051 | Unidentified | - | | - | | ++ | + | | + | | - | | - | | - | | - | |  |
| HQA052 | *Streptomyces gougerotii* NBRC 3198^T^ (AB184742) (99.58) | - | | - | | + | ++ | | - | | - | | - | | - | | - | |  |
| HQA053 | Unidentified | - | | - | | +++ | + | | + | | - | | - | | - | | - | |  |
| HQA054 | *Streptomyces violascens* ISP 5183^T^ (AY999737) (98.32) | - | | - | | + | - | | + | | - | | - | | 391.01±5.24 | | 986.29±6.39 | |  |
| HQA055 | Unidentified | - | | - | | + | ++ | | - | | - | | - | | - | | - | |  |
| HQA056 | Unidentified | - | | - | | +++ | ++ | | +++ | | - | | - | | - | | - | |  |
| HQA057 | *Streptomyces violascens* ISP 5183^T^ (AY999737) (99.32) | - | | - | | ++ | + | | + | | - | | - | | - | | - | |  |
| HQA058 | Unidentified | - | | - | | +++ | ++ | | ++ | | - | | - | | - | | - | |  |
| HQB215 | *Bacillus altitudinis* MTCC 7306^T^ (AJ831842) (98.83) | - | | - | | - | - | | - | | - | | - | | - | | 678.21±10.34 | |  |
| HQB216 | *Bacillus altitudinis* MTCC 7306^T^ (AJ831842) (98.28) | - | | - | | - | - | | - | | - | | - | | 295.46±8.92 | | - | |  |
| HQB217 | *Bacillus altitudinis* MTCC 7306^T^ (AJ831842) (98.29) | - | | - | | - | - | | - | | - | | - | | - | | - | |  |
| HQB218 | *Bacillus altitudinis* MTCC 7306^T^ (AJ831842) (97.44) | - | | - | | - | - | | - | | - | | ++ | | - | | 206.47±3.69 | |  |
| HQB219 | *Bacillus tequilensis* KCTC 13622^T^ (LC038165) (99.71) | - | | - | | - | - | | - | | - | | - | | - | | - | |  |
| HQB220 | *Bacillus altitudinis* MTCC 7306^T^ (AJ831842) (100) | - | | - | | - | - | | - | | - | | - | | - | | 692.18±8.74 | |  |
| HQB221 | *Bacillus altitudinis* MTCC 7306^T^ (AJ831842) (97.70) | - | | - | | - | - | | - | | - | | - | | 734.48±7.81 | | 150.78±10.23 | |  |
| HQB222 | *Halomonas litopenaei* SYSU ZJ2214^T^ (KP301091) (99.71) | - | | - | | - | - | | - | | - | | - | | - | | - | |  |
| HQB223 | *Bacillus altitudinis* MTCC 7306^T^ (AJ831842) (97.67) | - | | - | | - | - | | - | | - | | + | | - | | 181.03±5.14 | |  |
| HQB224 | *Bacillus hwajinpoensis* KCCM 41641^T^ (AF541966) (98.42) | + | | - | | - | + | | - | | - | | +++ | | - | | - | |  |
| HQB225 | *Bacillus infantis* NRRL B-14911^T^ (JF495108) (98.42) | - | | - | | - | - | | - | | - | | - | | - | | 288.1±5.22 | |  |

TABLE S2 continued

| **Test strains** | **16S rRNA comparison results and similarity (%)** | | **Inhibition degree against indicator strains^a^** | | | | | | | | | | | | | **Antiproliferative activities IC_50_ (μg**·**mL^-1^) ^b^** | | | |
| --- | --- | --- | --- | --- | --- | --- | --- | --- | --- | --- | --- | --- | --- | --- | --- | --- | --- | --- | --- |
|  |  |  | **Ec** | | **Pa** | **Bs** | | **Sa** | | **Ca** | | **Va** | | **Vp** | | **Bel 7402** | | **HeLa** | |
| HQB226 | *Halomonas litopenaei* SYSU ZJ2214^T^ (KP301091) (99.85) | - | | - | | - | - | | - | | - | | - | | - | | 267.55±4.21 | |  |
| HQB227 | *Bacillus altitudinis* MTCC 7306^T^ (AJ831842) (98.67) | + | | - | | - | - | | - | | - | | - | | - | | 458.09±6.33 | |  |
| HQB228 | *Bacillus oceanisediminis* JCM 16506^T^ (GQ292772) (97.34) | - | | - | | - | - | | - | | - | | - | | - | | - | |  |
| HQB229 | *Bacillus aciditolerans* JCM 32973^T^(MG589508) (99.13) | - | | - | | - | - | | - | | - | | ++ | | - | | - | |  |
| HQB230 | *Bacillus altitudinis* MTCC 7306^T^ (AJ831842) (97.44) | - | | - | | - | - | | - | | - | | - | | - | | 260.71±7.25 | |  |
| HQB231 | *Bacillus altitudinis* MTCC 7306^T^ (AJ831842) (98.07) | - | | - | | - | - | | - | | - | | - | | - | | 155.03±5.45 | |  |
| HQB232 | *Bacillus altitudinis* MTCC 7306^T^ (AJ831842) (98.73) | - | | - | | - | - | | - | | - | | - | | - | | 205.97±4.86 | |  |
| HQB233 | *Bacillus cereus* ATCC 14579^T^ (AE016877) (96.90) | - | | - | | - | - | | - | | - | | - | | - | | 896.37±8.41 | |  |
| HQB234 | *Bacillus altitudinis* MTCC 7306^T^ (AJ831842) (99.93) | - | | - | | - | - | | - | | - | | - | | - | | 644.89±6.95 | |  |
| HQB235 | *Bacillus altitudinis* MTCC 7306^T^ (AJ831842) (97.11) | - | | - | | ++ | - | | - | | - | | - | | - | | 708.23±7.81 | |  |
| HQB236 | *Bacillus altitudinis* MTCC 7306^T^ (AJ831842) (98.42) | - | | - | | - | - | | - | | - | | - | | - | | - | |  |
| HQB237 | *Bacillus altitudinis* MTCC 7306^T^ (AJ831842) (98.83) | - | | - | | - | - | | - | | - | | - | | 0.44±0.09 | | - | |  |
| HQB238 | *Bacillus aryabhattai* MTCC 7902^T^ (EF114313) (100) | - | | - | | + | - | | - | | - | | - | | - | | - | |  |
| HQB239 | *Fictibacillus phosphorivorans* CCM 8426^T^ (JX258924) (99.03) | - | | - | | + | - | | - | | - | | - | | - | | 298.21±4.11 | |  |
| HQB240 | *Bacillus altitudinis* MTCC 7306^T^ (AJ831842) (99.24) | - | | - | | + | - | | - | | - | | - | | - | | - | |  |
| HQB241 | *Bacillus altitudinis* MTCC 7306^T^ (AJ831842) (98.15) | - | | - | | + | - | | - | | - | | - | | - | | - | |  |
| HQB242 | *Bacillus altitudinis* MTCC 7306^T^ (AJ831842) (98.56) | - | | - | | - | - | | - | | - | | - | | - | | 295.53±6.25 | |  |
| HQB243 | *Bacillus licheniformis* ATCC 14580^T^ (KY034369) (98.55) | - | | - | | - | - | | - | | + | | - | | 161.06±12.54 | | - | |  |
| HQB244 | *Bacillus altitudinis* MTCC 7306^T^ (AJ831842) (97.24) | - | | - | | - | - | | - | | - | | - | | - | | 261.23±7.77 | |  |
| HQB245 | *Bacillus altitudinis* MTCC 7306^T^ (AJ831842) (97.94) | - | | - | | + | - | | - | | - | | - | | - | | - | |  |
| HQB246 | *Bacillus velezensis* CECT 5686^T^ (AY603658) (99.43) | - | | - | | - | ++ | | ++ | | - | | - | | 187.83±10.28 | | 187.35±6.25 | |  |

TABLE S2 continued

| **Test strains** | **16S rRNA comparison results and similarity (%)** | | **Inhibition degree against indicator strains^a^** | | | | | | | | | | | | | **Antiproliferative activities IC_50_ (μg**·**mL^-1^) ^b^** | | | |
| --- | --- | --- | --- | --- | --- | --- | --- | --- | --- | --- | --- | --- | --- | --- | --- | --- | --- | --- | --- |
|  |  |  | **Ec** | | **Pa** | **Bs** | | **Sa** | | **Ca** | | **Va** | | **Vp** | | **Bel 7402** | | **HeLa** | |
| HQB247 | *Bacillus velezensis* CECT 5686^T^ (AY603658) (99.07) | - | | - | | - | - | | - | | - | | - | | 506.11±6.45 | | - | |  |
| HQB248 | *Bacillus infantis* NRRL B-14911^T^ (JF495108) (98.49) | - | | - | | - | +++ | | +++ | | - | | - | | - | | - | |  |
| HQB249 | *Bacillus altitudinis* MTCC 7306^T^ (AJ831842) (99.04) | - | | - | | - | - | | - | | - | | - | | - | | - | |  |
| HQB250 | *Citrobacter freundii* ATCC 8090^T^ (KM515969) (97.85) | - | | - | | - | - | | - | | - | | - | | 926.67±6.12 | | - | |  |
| HQB251 | *Bacillus velezensis* CECT 5686^T^ (AY603658) (99.21) | - | | - | | - | - | | +++ | | - | | - | | 417.54±4.03 | | 527.44±7.16 | |  |
| HQB252 | *Bacillus thermotolerans*  KACC 16706^T^ (MF359551) (96.85) | - | | - | | - | +++ | | ++ | | - | | - | | 263.21±3.10 | | 897.13±8.90 | |  |
| HQB253 | *Bacillus altitudinis* MTCC 7306^T^ (AJ831842) (99.24) | - | | - | | - | - | | + | | - | | - | | - | | - | |  |
| HQB254 | Unidentified | - | | - | | + | - | | - | | - | | + | | - | | - | |  |
| HQB256 | Unidentified | - | | - | | + | - | | - | | - | | - | | - | | - | |  |
| HQB262 | *Bacillus siamensis* KCTC 13613^T^ (KT781674) (99.61) | - | | - | | - | - | | - | | ++ | | - | | - | | - | |  |
| HQB268 | Unidentified | + | | + | | + | + | | - | | - | | - | | 331.7±5.11 | | 373.57±6.27 | |  |
| HQB617 | *Vibrio neocaledonicus*NC470^T^ (JQ934828) (99.80) | - | | - | | ++ | - | | - | | - | | - | | - | | 689.68±9.21 | |  |
| HQB618 | *Bacillus vietnamensis* JCM 11124^T^ (AB099708) (98.25) | - | | - | | - | - | | - | | - | | - | | - | | - | |  |
| HQB619 | Unidentified | - | | - | | - | - | | - | | - | | - | | - | | - | |  |
| HQB620 | Unidentified | - | | - | | - | - | | - | | - | | - | | - | | 453.36±3.02 | |  |
| HQB621 | *Shewanella loihica* DSM 17748^T^ (CP000606) (98.44) | - | | - | | - | - | | - | | - | | - | | - | | - | |  |
| HQB622 | *Vibrio hyugaensis* LMG 28466^T^ (LC004912) (99.88) | - | | - | | - | - | | - | | - | | - | | - | | - | |  |
| HQB623 | Unidentified | - | | - | | - | - | | - | | - | | - | | - | | - | |  |
| HQB624 | *Bacillus siamensis* KCTC 13613^T^ (KT781674) (99.34) | - | | - | | - | - | | + | | ++ | | - | | - | | 104.04±4.29 | |  |
| HQB625 | Unidentified | - | | - | | - | - | | - | | - | | - | | - | | - | |  |
| HQB626 | *Salinicoccus roseus* DSM 5351^T^ (X94559) (98.70) | - | | - | | - | - | | - | | - | | - | | - | | - | |  |

TABLE S2 continued

| **Test strains** | **16S rRNA comparison results and similarity (%)** | | **Inhibition degree against indicator strains^a^** | | | | | | | | | | | | | **Antiproliferative activities IC_50_ (μg**·**mL^-1^) ^b^** | | | |
| --- | --- | --- | --- | --- | --- | --- | --- | --- | --- | --- | --- | --- | --- | --- | --- | --- | --- | --- | --- |
|  |  |  | **Ec** | | **Pa** | **Bs** | | **Sa** | | **Ca** | | **Va** | | **Vp** | | **Bel 7402** | | **HeLa** | |
| HQB627 | Unidentified | - | | - | | - | - | | - | | - | | - | | - | | 254.68±3.86 | |  |
| HQB628 | *Tenacibaculum lutimaris* DSM 16505^T^ (AY661691) (98.32) | - | | - | | + | - | | - | | - | | - | | - | | 339.46±6.85 | |  |
| HQB629 | Unidentified | - | | - | | - | - | | - | | - | | - | | - | | 910.64±6.56 | |  |
| HQB630 | *Vibrio neocaledonicus* NC470^T^ (JQ934828) (98.42) | - | | - | | - | - | | - | | - | | - | | - | | - | |  |
| HQB631 | *Fictibacillus halophilus* MCC 2765^T^ (KP265300) (99.89) | - | | - | | - | ++ | | - | | - | | - | | - | | 896.22±9.34 | |  |
| HQB632 | *Vibrio neocaledonicus* NC470^T^ (JQ934828) (98.24) | - | | - | | ++ | + | | - | | - | | - | | - | | - | |  |
| HQB633 | Unidentified | - | | - | | - | - | | - | | - | | - | | - | | 941.32±7.63 | |  |
| HQB634 | *Bacillus marisflavi* JCM 11544^T^ (DQ105973) (96.90) | - | | - | | ++ | + | | - | | - | | - | | - | | 946.24±5.90 | |  |
| HQB635 | *Kocuria polaris* MTCC 3702^T^ (AJ278868) (99.29) | - | | - | | - | - | | - | | - | | - | | - | | - | |  |
| HQB636 | *Citricoccus nitrophenolicus* DSM 23311^T^(GU797177) (99.l0) | ++ | | - | | +++ | ++ | | - | | +++ | | - | | - | | - | |  |
| HQB637 | Unidentified | - | | - | | - | - | | - | | - | | - | | - | | - | |  |
| HQB638 | Unidentified | - | | - | | - | - | | - | | - | | - | | - | | - | |  |
| HQB639 | Unidentified | + | | - | | - | - | | + | | - | | - | | - | | - | |  |
| HQB640 | *Bacillus firmus* NBRC 15306^T^ (X60616) (99.21) | - | | - | | + | - | | - | | - | | - | | - | | 876.87±4.93 | |  |
| HQB641 | Unidentified | - | | - | | + | + | | + | | ++ | | - | | - | | - | |  |
| HQB642 | *Bacillus safensis* subsp. *safensis* FO-36b^T^ (MK424279) (98.93) | - | | - | | - | - | | - | | ++ | | ++ | | - | | - | |  |
| HQB643 | *Bacillus zhangzhouensis* LMG 27144^T^ (JX680133) (99.58) | - | | - | | ++ | ++ | | - | | - | | - | | - | | - | |  |
| HQB644 | Unidentified | - | | - | | - | - | | - | | - | | - | | - | | 846.78±9.34 | |  |
| HQB645 | Unidentified | - | | - | | - | + | | - | | - | | - | | - | | - | |  |
| HQB646 | Unidentified | - | | - | | - | - | | ++ | | - | | - | | 180.78±6.23 | | 113.97±7.32 | |  |
| HQB647 | Unidentified | - | | - | | - | - | | - | | - | | - | | - | | - | |  |

TABLE S2 continued

| **Test strains** | **16S rRNA comparison results and similarity (%)** | | **Inhibition degree against indicator strains^a^** | | | | | | | | | | | | | **Antiproliferative activities IC_50_ (μg**·**mL^-1^) ^b^** | | | |
| --- | --- | --- | --- | --- | --- | --- | --- | --- | --- | --- | --- | --- | --- | --- | --- | --- | --- | --- | --- |
|  |  |  | **Ec** | | **Pa** | **Bs** | | **Sa** | | **Ca** | | **Va** | | **Vp** | | **Bel 7402** | | **HeLa** | |
| HQB648 | Unidentified | - | | - | | - | - | | - | | - | | - | | - | | - | |  |
| HQB649 | *Bacillus siamensis* KCTC 13613^T^ (KT781674) (99.41) | - | | - | | + | - | | - | | - | | - | | - | | - | |  |
| HQB650 | *Paenibacillus kribbensis* JCM 11465^T^ (AF391123) (96.72) | - | | - | | - | - | | - | | - | | - | | 343.61±7.49 | | 218.64±7.19 | |  |
| HQB651 | *Bacillus infantis* NRRL B-14911^T^ (JF495108) (98.80) | - | | - | | - | - | | +++ | | - | | - | | - | | - | |  |
| HQB652 | *Bacillus megaterium* NBRC 15308^T^ (AB271751) (98.94) | - | | - | | - | - | | - | | - | | - | | - | | - | |  |
| HQB653 | Unidentified | ++ | | - | | - | - | | - | | - | | - | | 179.73±3.57 | | 213.13±4.55 | |  |
| HQB654 | Unidentified | - | | - | | + | - | | - | | - | | - | | - | | - | |  |
| HQB655 | Unidentified | - | | - | | - | - | | - | | - | | - | | - | | 765.47±7.89 | |  |
| HQB656 | *Rhodococcus hoagii* DSM 20295^T^ (X82052) (97.24) | - | | - | | - | - | | - | | - | | - | | - | | - | |  |
| HQB657 | Unidentified | - | | - | | - | - | | - | | - | | - | | - | | - | |  |
| HQB658 | *Bacillus frigoritolerans* DSM 8801^T^ (AM747813) (99.65) | - | | - | | - | - | | - | | - | | - | | - | | - | |  |
| HQB659 | *Bacillus flexus* NBRC 15715^T^ (FJ584305) (99.28) | - | | - | | - | - | | - | | - | | - | | - | | - | |  |
| HQB660 | *Bacillus tequilensis* KCTC 13622^T^ (LC038165) (99.30) | - | | - | | - | - | | - | | - | | - | | - | | 965.87±6.94 | |  |
| HQB661 | Unidentified | - | | - | | + | - | | - | | - | | - | | - | | - | |  |
| HQB662 | Unidentified | - | | - | | - | - | | - | | - | | - | | - | | - | |  |
| HQB663 | *Bacillus subtilis* subsp. *spizizenii* NRRL B-23049^T^ ( AF074970) (99.23) | - | | - | | - | ++ | | +++ | | - | | - | | - | | 308.37±4.03 | |  |
| HQB664 | Unidentified | - | | - | | - | + | | - | | - | | - | | - | | - | |  |
| HQB665 | Unidentified | - | | - | | + | - | | - | | - | | - | | - | | - | |  |
| HQB666 | Unidentified | - | | - | | + | - | | - | | - | | - | | - | | 207.91±5.25 | |  |
| HQB667 | Unidentified | - | | - | | - | - | | ++ | | - | | - | | 291.69±11.34 | | 129.77±5.28 | |  |

TABLE S2 continued

| **Test strains** | **16S rRNA comparison results and similarity (%)** | | **Inhibition degree against indicator strains^a^** | | | | | | | | | | | | | **Antiproliferative activities IC_50_ (μg**·**mL^-1^) ^b^** | | | |
| --- | --- | --- | --- | --- | --- | --- | --- | --- | --- | --- | --- | --- | --- | --- | --- | --- | --- | --- | --- |
|  |  |  | **Ec** | | **Pa** | **Bs** | | **Sa** | | **Ca** | | **Va** | | **Vp** | | **Bel 7402** | | **HeLa** | |
| HQB668 | Unidentified | - | | - | | - | - | | - | | - | | - | | 756.31±9.65 | | 687.48±8.12 | |  |
| HQB669 | Unidentified | - | | - | | - | - | | ++ | | - | | - | | - | | - | |  |
| HQB670 | *Bacillus hwajinpoensis* KCCM 41641^T^ (AF541966) (98.74) | - | | - | | - | ++ | | +++ | | - | | - | | 400.92±4.50 | | - | |  |

^a^ Antimicrobial activities were tested against *Escherichia coli* (Ec), *Pseudomonas aeruginosa* (Pa), *Bacillus subtilis* (Bs), *Staphylococcus aureus* (Sa), *Candida albicans* (Ca), *Vibrio anguillarum* (Va) and *Vibrio parahaemolyticus* (Vp).

Symbols of inhibition degree against indicator strains: (-), no inhibition; (+), 11 < inhibition zone < 13 mm; (++), 13 ≤ inhibition zone < 16 mm; (+++), 16 ≤ inhibition zone < 22 mm; (++++), inhibition zone ≥ 22 mm.

^b^ Data is expressed as mean ± SE of three or more experiments. The symbol (-) means that the crude extract of test strains at a concentration of 1 mg·mL^-1^ showed less than 50% growth inhibition of the tumor cells.
